# Neuropathic Pain: From Mechanisms to Therapeutic Interventions

**DOI:** 10.1002/mco2.71061

**Published:** 2026-10-13

**Authors:** Yaling Dai, Mengquan Tan, Man Yuan, Huixian Zhou, Qin Xie, Jiling Zeng, Yixin Yang, Xinran He, Siyuan Song

**Affiliations:** ^1^ Fujian Key Laboratory of Aptamers Technology, Fuzhou General Teaching Hospital (The 900th Hospital) Fujian University of Traditional Chinese Medicine Fuzhou China; ^2^ The Institution of Rehabilitation Industry Fujian University of Traditional Chinese Medicine Fuzhou China; ^3^ Eye Center, Renmin Hospital of Wuhan University Wuhan University Wuhan China; ^4^ Department of Internal Medicine, Montefiore Medical Center Albert Einstein College of Medicine New York New York USA; ^5^ Department of Family Medicine Good Samaritan Hospital San Jose California USA; ^6^ Department of Internal Medicine Nazareth Hospital Philadelphia Pennsylvania USA; ^7^ Sun Yat‐sen Cancer Center Guangzhou China; ^8^ Department of Anesthesiology Boston University Boston Massachusetts USA; ^9^ Department of Anesthesiology, Montefiore Medical Center Albert Einstein College of Medicine New York New York USA; ^10^ Department of Neuroscience Baylor College of Medicine Houston Texas USA

**Keywords:** clinical stratification, mechanism‐guided treatment, multimodal imaging, neuroinflammation, neuropathic pain, pain circuits

## Abstract

Neuropathic pain is caused by injury or disease of the somatosensory system. It involves peripheral nerve injury, central sensitization, transmitter imbalance, glial activation, and large‐scale network remodeling. Despite substantial advances in pain biology, clinical assessment continues to rely predominantly on patient‐reported symptoms, sensory examination, and conventional neurophysiological testing, approaches that often fail to delineate the predominant biological mechanisms underlying pain in individual patients. Recent advances in sensory phenotyping and multimodal imaging have enabled in vivo characterization of abnormalities at the receptor, neuroinflammatory, and circuit levels, thereby providing a foundation for mechanism‐based stratification and more individualized therapeutic decision‐making. This review summarizes neuropathic pain from clinical stratification to mechanism‐based imaging and therapeutic intervention. We describe key clinical dimensions, including sensory gain, sensory loss, peripheral and central pain generators, mixed nociceptive‐neuropathic pain, central sensitization, and autonomic or small‐fiber modifiers. These dimensions help explain why patients with the same diagnosis may show different symptoms and treatment responses. We also review major molecular mechanisms, including glutamatergic overactivity, GABAergic and glycinergic disinhibition, monoaminergic remodeling, and neuroimmune modulation. These changes reshape spinal, thalamocortical, limbic, salience, reward, cerebellar, and descending control circuits. Peripheral nerve ultrasound and MR neurography can help identify structural peripheral pain generators. PET, MRS, MRI, DTI, fMRI, EEG, and multimodal imaging can assess receptor availability, neurotransmitter balance, neuroinflammation, white matter integrity, and network connectivity. These imaging tools may link clinical phenotypes with biological mechanisms and help define mechanism‐based subtypes. Imaging biomarkers may also support treatment selection, treatment monitoring, neuromodulation targeting, and translational trial design. This framework supports a shift from symptom‐based assessment to mechanism‐guided precision care for neuropathic pain.

## Graphical abstract

Neuropathic pain arises from peripheral nerve injury, central sensitization, neurotransmitter imbalance, neuroimmune activation, and maladaptive circuit remodeling. Clinical phenotyping and multimodal imaging can identify dominant peripheral and central mechanisms. Linking sensory profiles, molecular and circuit biomarkers, and treatment response supports mechanism‐guided pharmacotherapy, neuromodulation, rehabilitation, and precision management.

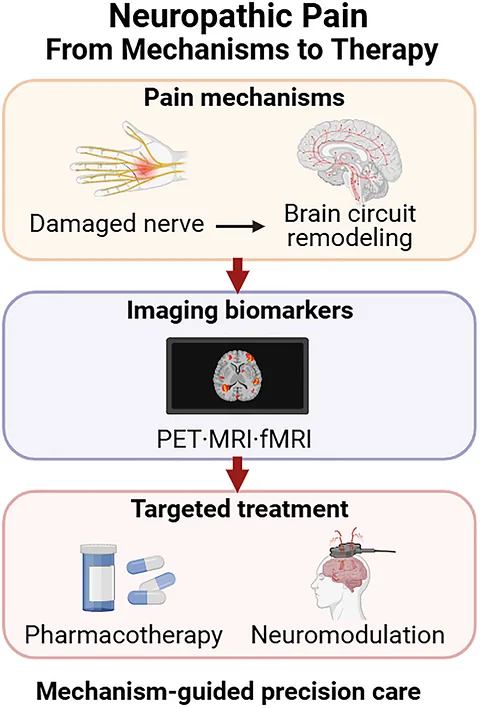

## 1 Introduction

Neuropathic pain is pain that arises because the somatosensory system has been injured or diseased. It is among the most disabling chronic pain conditions, and it reflects contributions from both peripheral generators and central maintenance processes [1]. Typical clinical settings include diabetes‐related neuropathy, postherpetic neuralgia, trigeminal neuralgia, phantom limb pain after amputation, and central pain after stroke. Across populations, prevalence estimates vary by cause, yet surveys of the general adult population consistently place neuropathic pain in the single‐digit percentage range, with a meaningful fraction reporting moderate to severe symptoms [2]. Rates are higher in older adults and in women, and syndromes such as lumbar radiculopathy and lower limb neuropathic pain are especially common [3, 4].

The disease burden associated with neuropathic pain extends far beyond the intensity of the pain itself. Patients often experience sleep disturbances, anxiety and depression, as well as cognitive complaints such as impaired attention and memory function [5]. These comorbidities not only exacerbate functional disability but also significantly reduce quality of life. Health economics research further indicates that healthcare resource utilization among patients with neuropathic pain is substantially higher than that among patients with nonneuropathic chronic pain [6]. Therefore, clinical practice urgently needs to shift from a symptom management approach to mechanism‐oriented objective diagnosis and precision treatment.

Unlike inflammatory or nociceptive pain, neuropathic pain involves nervous system remodeling itself in a pathological way [7]. This starts at the periphery, where nociceptors and dorsal root ganglion (DRG) neurons develop lasting changes in how they express ion channels and how they fire [8]. For instance, alterations in voltage‐gated sodium channels such as Nav1.7, Nav1.8, and Nav1.9 lead to ectopic firing of neurons [9]. Subsequently, in the spinal dorsal horn and higher brain regions, including the thalamus and cortex, incoming excitatory signals become persistently amplified, while inhibitory modulation is progressively attenuated [10]. Furthermore, activated microglia and astrocytes establish a vicious positive feedback loop with neurons through P2X4 and P2X7 receptors, as well as inflammatory cytokines and neurotrophic factors [11]. This mechanism converts pain from an initial acute nociceptive signal into an autonomous neural network disorder. Within the local circuits of the spinal dorsal horn, inhibitory interneurons exhibit diminished function, leading to excessive firing activity of projection neurons [12]. This boosts the ascending pain signals traveling up to the thalamus and cortex. Then, the cortex and thalamus turn around and amplify the incoming peripheral input even more through descending facilitatory pathways [13]. This creates a positive feedback loop that goes from the spinal cord to the thalamus to the cortex and back down through the facilitatory pathways, which keeps pain signals reinforced and spread out over time [14].

Currently, the diagnosis and assessment of neuropathic pain still rely primarily on patients’ subjective descriptions, screening questionnaires, and neurophysiological examinations (such as nerve conduction velocity and quantitative sensory testing) [15]. While these tools have some value in case detection and sensory profiling, they suffer from limited specificity, poor reproducibility, and difficulty in distinguishing pain subtypes with different underlying mechanisms. More importantly, they are unable to directly reflect the central molecular changes and neural network dysfunction that sustain pain, thereby limiting the ability to achieve precise diagnosis [16]. In recent years, neuroimaging techniques have provided powerful tools for revealing the central mechanisms underlying neuropathic pain. Positron emission tomography and single‐photon emission computed tomography allow for in vivo quantitative detection of receptor availability, transporter function, and neuroinflammatory signals. Magnetic resonance imaging methods can assess neurotransmitter concentrations, patterns of brain activation, white matter fiber integrity, and regional changes in gray matter volume [17]. A large body of research has shown that neuropathic pain involves extensive remodeling of the glutamatergic, GABAergic [18], serotonergic [19], dopaminergic [20], and noradrenergic systems [21] and is closely associated with dysfunction in thalamocortical, limbic, and descending modulatory pathways. Because the basis of neuropathic pain is no longer simply inflammation or tissue damage, conventional nonsteroidal anti‐inflammatory drugs (NSAIDs) and opioid analgesics generally have limited efficacy in neuropathic pain, and long‐term use is often constrained by tolerance, adverse effects, and the risk of addiction [22]. Although current guidelines place greater emphasis on treatments such as calcium channel modulators, serotonin–norepinephrine reuptake inhibitors, and neuromodulation, a considerable proportion of patients still remain in a state of partial remission or even refractory under standard care [23]. This suggests an urgent need to develop more precise targeted drugs and biologics aimed at key nodes, such as different molecular markers and central inhibitory circuits.

This review aims to integrate advances in molecular imaging and systems‑level neural circuit research, propose an imaging‑based framework for neuropathic pain, and explore translational pathways from mechanisms to therapeutic interventions. From a clinical perspective, we classify neuropathic pain into subtypes and summarize sensory phenotypes, peripheral and central pain generators, mixed pain, and overlapping modulating factors. We then introduce peripheral and central imaging methods, with a focus on peripheral nerve ultrasound, magnetic resonance neurography, and central molecular and network imaging techniques. Furthermore, we discuss how to move from imaging biomarkers to therapeutic interventions, including mechanism‑guided treatment selection and treatment monitoring. By linking molecular biology, neural circuit function, and clinical outcomes, we aim to provide a systematic theoretical framework and practical reference for the mechanistic understanding and precise intervention of neuropathic pain.

## 2 Clinical Stratification of Neuropathic Pain

### 2.1 Sensory Phenotypes and Symptom Profiles

Neuropathic pain should be classified primarily based on its clinical features, not according to the disease name [24, 25, 26]. This is necessary. Patients with neuropathic pain often show different symptoms, which may reflect different causes. Some patients feel burning pain, electric shock‐like pain, or shooting pain; this type of pain may appear when injured nerve pathways become too active [27]. In some other patients, pain is caused by light touch or cold stimulation, which may mean that pain pathways in the central nervous system have become more sensitive. Additionally, there are some patients who mainly have numbness, together with a reduced feeling of heat or cold; this usually suggests that part of the nerve function has been lost [24, 25, 28].

Quantitative sensory testing and sensory examination at the bedside support this approach [29, 30]. Previous large‐scale studies have reported that peripheral neuropathic pain can be divided into sensory loss, thermal hyperalgesia, and mechanical hyperalgesia profiles [24, 25]. These profiles are not just descriptive but are likely symptomatic of different combinations of peripheral nociceptor damage, ectopic firing, disinhibition, and central sensitization [24, 26, 27]. A patient with marked thermal or mechanical gain may indicate retained but sensitized nociceptive pathways, while a patient with marked sensory loss may represent more deafferentation and decreased peripheral sensation, with central amplification contributing in some patients.

A patient's sensory phenotype can also help explain why patients in the same disease group may respond differently to treatment [31, 32, 33]. Patients with diabetic neuropathy, postherpetic neuralgia, or radiculopathy can all have neuropathic pain, but the pain mechanisms behind them may not be the same [24, 25, 26, 27].

### 2.2 Peripheral Versus Central Pain Generators

A second and clinically important level of neuropathic pain classification concerns the site of the pain generator, that is, whether the dominant process is peripheral or central [27, 34, 35]. Current definitions describe neuropathic pain as pain caused by a lesion or disease of the somatosensory nervous system, yet the anatomical level of that lesion still matters [34, 35]. When the pathological process involves the peripheral nerve, dorsal root ganglion, nerve root, or plexus, the condition is usually regarded as peripheral neuropathic pain; painful diabetic neuropathy, postherpetic neuralgia, postsurgical nerve injury, and entrapment neuropathy are typical examples. Central neuropathic pain refers to a different situation in which the lesion is located within the brain or spinal cord, as seen in poststroke pain, spinal cord injury pain, or pain associated with multiple sclerosis [34, 35, 36].

Common biological processes occur differently at the periphery and central levels [27, 36]. Pathophysiological changes in peripheral neuropathic pain are primarily related to axonal injury, aberrant membrane excitability, ectopic discharge, ion‐channel remodeling, inflammatory mediator secretion, and altered neuron–immune crosstalk around the injured nerve [27, 37]. Central neuropathic pain, however, involves a greater role for loss of sensory input, aberrant synaptic plasticity, a reduction in descending inhibitory mechanisms, and long‐term remodeling [27, 36]. Nevertheless, these two levels should not be dichotomized. Although peripheral lesioning can be the cause of the disease, in some patients, central amplification mechanisms can become major contributors to the persistence of pain [27]. Hence, peripheral and central generators of pain can be regarded as two levels of the same disease.

This distinction also matters for imaging and for monitoring treatment response [27, 38, 39, 40]. When the main pain generator is peripheral, nerve‐focused imaging, electrodiagnostic testing, or treatment directed at the lesion may be more informative. When the dominant generator is central, the focus usually shifts. Molecular imaging, structural MRI, diffusion imaging, and functional network imaging may better explain persistent symptoms and different treatment responses. Many patients do not stay at one point on this peripheral–central spectrum over time. In early disease, pain may still follow peripheral pathology more closely; in chronic disease, pain intensity may become less related to the size of the original lesion and more related to central sensitization and circuit‐level dysfunction [27, 36].

### 2.3 Mixed Nociceptive–Neuropathic Pain and Musculoskeletal Contributors

More commonly, neuropathic and nociceptive mechanisms coexist in “mixed pain”, that is, pain arising from both neuropathic and nociceptive mechanisms that may also mutually amplify each other [41]. This is the case, for example, for chronic low back pain with radicular features, chronic postsurgical pain with nerve involvement, entrapment syndromes, and some chronic musculoskeletal disorders with pain and central sensitization. The neuropathic mechanisms associated with somatosensory nerve injury and/or disease coexist with nociceptive mechanisms related to sustained tissue load, joint dysfunction, hyperactive muscles, fascia‐related pain, or other musculoskeletal causes [41, 42].

Musculoskeletal pathology may contribute to neuropathic pain through several related, mainly mechanical, pathways. In entrapment neuropathies, a peripheral nerve may be compressed or irritated as it passes through a narrow anatomical space [43]. Such compression can impair intraneural microcirculation, disturb axonal transport, and gradually change the structure of nerve fibers [44]. Peripheral nerves may also be exposed to tensile, shear, and compressive stress during posture change, limb movement, or repeated mechanical loading; as a result, nerve gliding may be reduced, and local mechanical irritation may become more obvious [45].

Mechanical stress can induce inflammation in proximity to the nerve. In animal experiments, even mild compression induces immune inflammation not only at the site of compression but also in the nearby dorsal root ganglion [46]. Cross‐talk between peripheral nerves and the immune system can sensitize nociceptors and potentially contribute to persistent pain after nerve injury [47]. Myofascial pain is largely classified within the nociceptive pain spectrum. Nevertheless, it can coincide with neuropathic pain, introducing an additional peripheral source of symptoms among patients experiencing mixed pain conditions [42]. While patients present symptoms with radiating pain, paresthesia, allodynia, or sensory loss with focal neurological signs, clinicians should investigate myofascial dysfunction, postural imbalance, repetitive loading, or nerve entrapment.

### 2.4 Overlapping Modifiers in Neuropathic Pain: Central Sensitization and Autonomic Traits

Central sensitization should be considered a modifying factor in neuropathic pain rather than a new term that supersedes neuropathic pain diagnosis [27, 48]. In this context, ongoing abnormal afferent input or a prior somatosensory injury may lead to a persistent state of exaggerated central nervous system amplification. Clinically, this may be seen as increased temporal summation, pain extending beyond the original lesion area, greater sensitivity to weak sensory input, or a clear mismatch between the structural lesion and the present level of pain. These findings do not make the neuropathic diagnosis invalid. Rather, they suggest that the current pain state is being supported not only by the original lesion but also by altered processing in the spinal cord and higher brain regions.

Autonomic features should also be considered clinically relevant modifiers, particularly in disorders that affect small somatic fibers together with autonomic fibers [49, 50]. A good example is small‐fiber neuropathy. In addition to burning pain, dysesthesia, and altered temperature sensation, patients can experience a spectrum of autonomic symptoms, including orthostatic intolerance, vasomotor symptoms, gastrointestinal symptoms, secretomotor symptoms, and heat intolerance. This is relevant to the discussion because it challenges us to consider the symptoms beyond pain and how these patients may have symptoms consistent with altered autonomic regulation in addition to altered pain signaling. Even if pain is predominant, autonomic symptoms may help to explain transient symptomatology or fluctuations, skin color changes, and severe discomfort when standing and in heat [49, 50].

Central sensitization and autonomic involvement make it difficult to estimate neuropathic pain severity from the lesion site alone [27, 48, 49, 50]. This point is clinically important. Two patients may have a similar peripheral nerve disorder, but their pain distribution, overall symptom burden, and daily functional impairment can still differ greatly. One reason is that central amplification may be stronger in one patient, while autonomic dysfunction may be more evident in another. These factors therefore have value for precision‐oriented phenotyping because they help describe differences that cannot be explained by lesion anatomy alone. They also connect naturally with imaging studies. Central sensitization and autonomic dysregulation can be examined indirectly through network‐level and molecular readouts rather than being assessed only through symptom questionnaires [27, 40].

When present, migraine‐related features should also be noted in the phenotyping process. Migraine itself is not classified as neuropathic pain, but migraine‐associated cutaneous allodynia, sensory hypersensitivity, and autonomic symptoms may indicate a stronger component of central amplification in some patients [51, 52]. In the migraine literature, cutaneous allodynia is commonly regarded as a clinical sign of central sensitization, and it has also been linked to migraine chronification [51]. Autonomic symptoms are frequently described as well; autonomic testing studies have reported altered autonomic function in some patients with migraine [53]. Flammer‐type features should be handled more cautiously. They are better recorded as autonomic or vascular modifiers, rather than as direct evidence of neuropathic pain. Flammer syndrome refers to a phenotype of primary vascular dysregulation, with associated symptoms or signs such as cold extremities, low blood pressure, altered drug sensitivity, increased pain sensitivity, and a tendency toward migraine (Figure 1) [54, 55].

**FIGURE 1:**
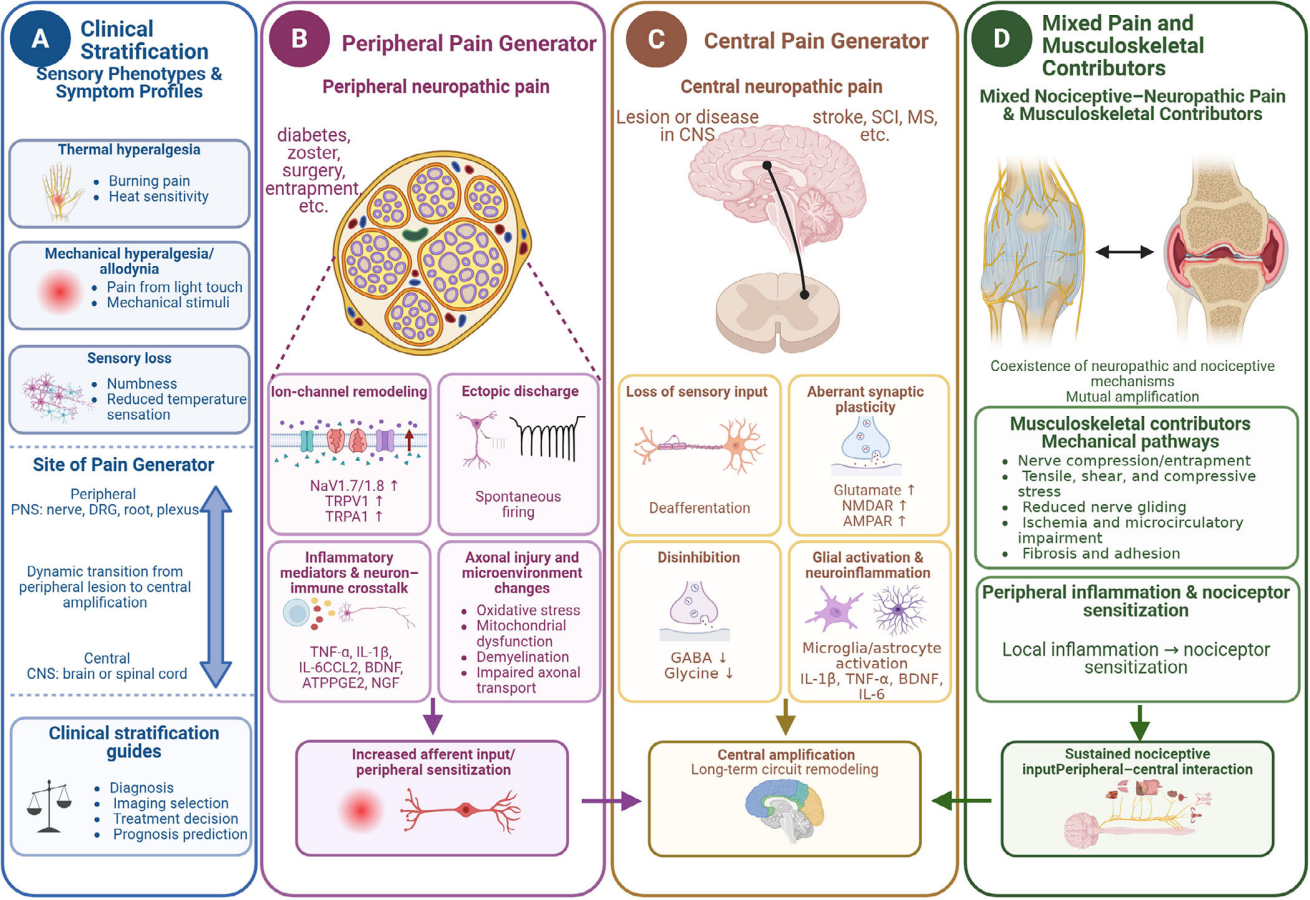
Clinical stratification of neuropathic pain integrates sensory phenotypes, pain‐generator location, and musculoskeletal contributors. (A) Sensory phenotypes include thermal hyperalgesia, mechanical hyperalgesia or allodynia, and sensory loss; these patterns can support clinical diagnosis, imaging selection, treatment decisions, and prognostic assessment. (B) Peripheral neuropathic pain may arise from nerve, dorsal root ganglion, root, or plexus pathology and is associated with ion‐channel remodeling, ectopic discharge, inflammatory mediators, and axonal injury. (C) Central neuropathic pain follows a lesion or disease affecting the brain or spinal cord and involves deafferentation, disinhibition, aberrant synaptic plasticity, and glial activation. (D) Mixed pain reflects the coexistence of neuropathic and nociceptive mechanisms, in which nerve entrapment, mechanical loading, local inflammation, and impaired nerve gliding may sustain peripheral input and central amplification. ATP, adenosine triphosphate; BDNF, brain‐derived neurotrophic factor; CCL2, C–C motif chemokine ligand 2; CNS, central nervous system; DRG, dorsal root ganglion; GABA, γ‐aminobutyric acid; IL‐1β, interleukin‐1 beta; IL‐6, interleukin‐6; MS, multiple sclerosis; NaV1.7 and NaV1.8, voltage‐gated sodium channel subtypes 1.7 and 1.8; NGF, nerve growth factor; NMDAR, N‐methyl‐D‐aspartate receptor; PGE2, prostaglandin E2; PNS, peripheral nervous system; SCI, spinal cord injury; TNF‐α, tumor necrosis factor alpha; TRPA1, transient receptor potential ankyrin 1; TRPV1, transient receptor potential vanilloid 1. Created with BioRender.com.

## 3 Molecular Mechanisms Relevant to Neuropathic Pain Phenotypes

Distinct forms of neuropathic pain develop due to intertwined molecular events: neurons become overexcited, inhibitory signaling fails, and maladaptive neuromodulation alongside immune responses take place simultaneously. These biological shifts interact closely rather than acting independently. Enhanced glutamatergic signaling boosts signal conduction. Meanwhile, insufficient inhibition from γ‐aminobutyric acid (GABA) and glycine systems reduces the barrier, enabling these signals to spread within neural pathways. Modifications to serotonin, dopamine, and norepinephrine‐related signaling pathways exert secondary effects on descending pain modulation, emotional perception, and glial cell behavior. Collectively, such molecular irregularities lay a biological foundation to interpret the anomalous imaging features identified within interconnected pain neural networks.

### 3.1 Glutamatergic Dysregulation

Glutamatergic dysregulation represents an important molecular change in neuropathic pain. After peripheral nerve injury, glutamate signaling tends to become stronger, and its clearance is also less effective [27, 56]. One possible explanation is the reduced uptake of glutamate by astrocytes; extracellular glutamate can then accumulate and extend beyond the synaptic cleft. As a result, peri‐ and extrasynaptic receptors may be exposed to more sustained glutamatergic stimulation, with prolonged postsynaptic excitation following this change. At the same time, dorsal horn projection neurons and excitatory interneurons become more responsive to the signal; spinal pain transmission is therefore strengthened, and nociceptive amplification can occur more readily.

An important part of this glutamatergic process is the increased involvement of GluN2B‐containing N‐methyl‐D‐aspartate (NMDA) receptors. Compared with other NMDA receptor forms, GluN2B‐rich receptors permit stronger and more prolonged calcium influx, which can favor the activation of calcium‐dependent kinases and transcriptional programs related to central sensitization [56]. α‐Amino‐3‐hydroxy‐5‐methyl‐4‐isoxazolepropionic acid (AMPA) receptor trafficking is also changed downstream of this signal. More glutamate ionotropic receptor AMPA type subunit 1 (GluA1)‐containing receptors are inserted into synapses, and in some settings, glutamate ionotropic receptor AMPA type subunit 2 (GluA2)‐lacking calcium‐permeable AMPA receptors may also increase [56, 57]. These receptor changes make synapses more excitable and make activity‐dependent potentiation easier to maintain. Metabotropic glutamate receptors add another layer to this process. In particular, mGluR5 can link glutamatergic activity with intracellular calcium release, extracellular signal‐regulated kinase (ERK) signaling, cAMP response element‐binding protein (CREB) activation, and plasticity‐related gene expression, producing a slower but more sustained form of synaptic gain [50, 56].

Glutamatergic aberrations may also underlie the observation that neuropathic pain is associated not only with sensory amplification but also with abnormal affect and cognition [27, 40]. Excessive glutamatergic drive is not confined to the dorsal horn [27, 56]. Likewise, aberrations may occur in supraspinal regions such as the thalamus, insula, anterior cingulate cortex, and hippocampus. This is relevant because it allows for linking the molecular and imaging phenotypes [40]. Increased mGluR5 availability, altered local glutamate‐GABA balance, and enhanced thalamo‐cortical or limbic coupling can be considered downstream manifestations (Figure 2).

**FIGURE 2:**
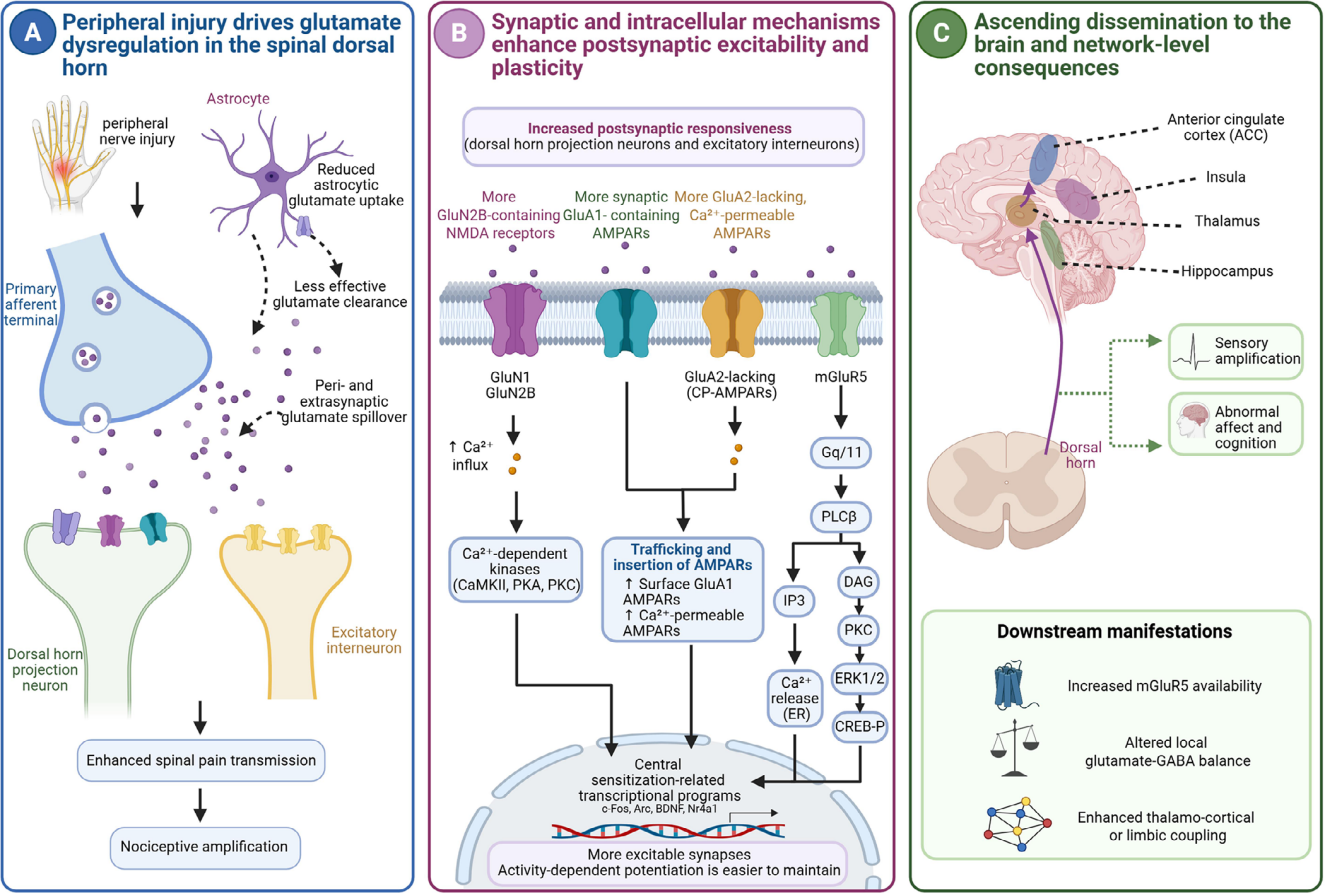
Peripheral nerve injury promotes glutamate‐driven spinal sensitization and supraspinal network dysfunction in neuropathic pain. (A) Peripheral nerve injury reduces astrocytic glutamate uptake, allowing extracellular glutamate to accumulate and extend to peri‐ and extrasynaptic sites in the dorsal horn. (B) Sustained glutamatergic signaling increases postsynaptic excitability through GluN2B‐containing NMDARs, altered AMPAR trafficking, and mGluR5‐dependent intracellular signaling, thereby facilitating central sensitization‐related transcriptional programs and synaptic potentiation. (C) Excessive ascending glutamatergic drive affects the thalamus, insula, ACC, and hippocampus and may be reflected by altered glutamate–GABA balance, increased mGluR5 availability, and enhanced thalamo‐cortical or limbic coupling. ACC, anterior cingulate cortex; AMPAR, α‐amino‐3‐hydroxy‐5‐methyl‐4‐isoxazolepropionic acid receptor; BDNF, brain‐derived neurotrophic factor; Ca2^+^, calcium ion; CaMKII, calcium/calmodulin‐dependent protein kinase II; c‐Fos, cellular proto‐oncogene FOS; CREB‐P, phosphorylated cAMP response element‐binding protein; DAG, diacylglycerol; ERK1/2, extracellular signal‐regulated kinase 1/2; GABA, γ‐aminobutyric acid; GluA1 and GluA2, glutamate ionotropic receptor AMPA type subunits 1 and 2; GluN1 and GluN2B, glutamate ionotropic receptor NMDA type subunits 1 and 2B; Gq/11, G protein alpha q/11; IP3, inositol 1,4,5‐trisphosphate; mGluR5, metabotropic glutamate receptor 5; NMDAR, N‐methyl‐D‐aspartate receptor; Nrg1, neuregulin 1; Nr4a1, nuclear receptor subfamily 4 group A member 1; PKA, protein kinase A; PKC, protein kinase C; PLCβ, phospholipase C beta. Created with BioRender.com.

### 3.2 GABAergic and Glycinergic Disinhibition

It is not only exuberant excitation that contributes to chronic neuropathic pain; deficient inhibition also plays a role. In the normal dorsal horn, GABAergic and glycinergic inhibitory interneurons prevent sensory neurons from conveying weak or innocuous signals to nociceptive projection neurons [27, 58]. After nerve injury, this inhibition is reduced [27, 58]. This loss may be due to the loss of inhibitory synapses, changes in the subunit composition of GABA_A_ receptors, a reduction in the synthesis of inhibitory neurotransmitters, and overall dysfunction of inhibitory interneurons in the dorsal horn. As these changes take place, both short‐lasting inhibition and background inhibitory control become weaker.

Another failure in inhibition contributing to this process is the loss of glycinergic inhibition. In particular, a reduction in the activity of glycine transporter 2 (GlyT2) results in lower levels of glycine at inhibitory synapses and reduced glycinergic inhibition in dorsal horn circuits [27, 58]. Disruptions in chloride homeostasis may also occur. For example, microglia‐derived factors, such as brain‐derived neurotrophic factor (BDNF), acting on dorsal horn neurons may reduce potassium‑chloride cotransporter 2 (KCC2) activity, resulting in a disruption of the chloride gradient and reduced effectiveness of GABAergic and glycinergic currents. The loss of inhibition is thought to allow sensory information that is normally filtered out to more readily activate nociceptive circuits.

Disinhibition may also account for why neuropathic pain can persist even after peripheral input has decreased. Once stable inhibition is lost in the dorsal horn and in higher‐order circuits, the system becomes more vulnerable to spontaneous activity, abnormal coupling between afferent channels, and the spread of excitation into supraspinal networks [27, 58]. This is why inhibitory markers are relevant in imaging‐based stratification. GABA‐related magnetic resonance spectroscopy measures, together with benzodiazepine‐site PET readouts, may provide indirect information about inhibitory function rather than simply reflecting nonspecific brain alterations (Figure 3) [40, 59].

**FIGURE 3:**
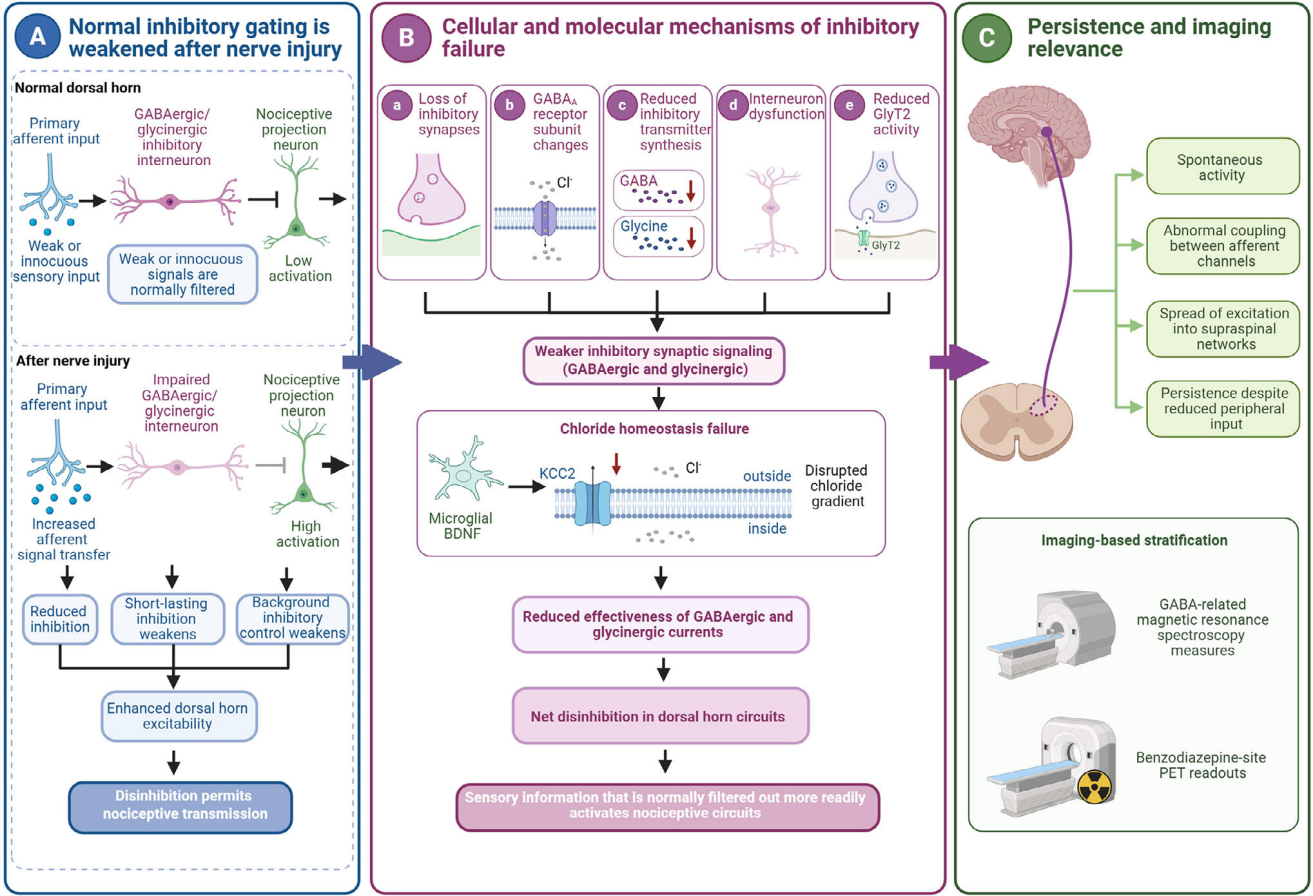
Loss of inhibitory gating permits nociceptive amplification and supports pain persistence after nerve injury. (A) In the normal dorsal horn, GABAergic and glycinergic interneurons filter weak or innocuous sensory input; after nerve injury, impaired inhibitory signaling permits stronger activation of nociceptive projection neurons. (B) Inhibitory failure is promoted by synaptic loss, altered GABA_A_ receptor composition, reduced GABA and glycine synthesis, interneuron dysfunction, and decreased GlyT2 activity. Microglia‐derived BDNF can further reduce KCC2 activity, disturb chloride homeostasis, and weaken GABAergic and glycinergic currents. (C) Persistent disinhibition may permit spontaneous activity, abnormal afferent coupling, and spread of excitation into supraspinal networks, while GABA‐related magnetic resonance spectroscopy and benzodiazepine‐site PET may provide indirect markers of impaired inhibition. BDNF, brain‐derived neurotrophic factor; Cl^−^, chloride ion; GABA, γ‐aminobutyric acid; GABA_A_ receptor, γ‐aminobutyric acid type A receptor; GlyT2, glycine transporter 2; KCC2, potassium‐chloride cotransporter 2; PET, positron emission tomography. Created with BioRender.com.

### 3.3 Serotonergic and Dopaminergic Remodeling

Serotonergic and dopaminergic remodeling helps place neuropathic pain beyond a purely sensory transmission disorder [27, 60]. Serotonin, in particular, does not act in one fixed direction across pain circuits; its effect depends on the receptor subtype involved, the anatomical target, and the state of the local circuit [60, 61]. In neuropathic pain, once descending control is altered and inhibitory restraint becomes weaker, serotonergic signaling may shift from an inhibitory role toward a facilitatory role [60, 62]. Under these conditions, serotonin is no longer only a brake on pain processing. In some circuits, it may instead strengthen pain facilitation and increase the affective salience of pain.

Dopamine is also part of this process. In chronic pain, mesolimbic and mesocorticolimbic dopamine signaling often declines, most clearly in circuits involved in reward, motivation, and behavioral adjustment [27, 63]. This change is not limited to mood. It may also influence the way pain is encoded and the way pain relief is assigned value. When dopaminergic tone becomes lower, reward sensitivity may decrease, and patients may find it harder to maintain adaptive behavior.

This monoaminergic perspective is also useful when imaging findings are interpreted. A reduction in dopaminergic synthesis capacity or lower dopamine receptor D2/3 (D2/3) receptor availability should not be read as an isolated imaging abnormality; these findings may point to a pain state in which negative affect and impaired motivation are more prominent [40, 63]. Altered serotonergic transporter or receptor signals need a similar reading. They may reflect changes in descending modulation, rather than only a broad or nonspecific chemical imbalance (Figure 4).

**FIGURE 4:**
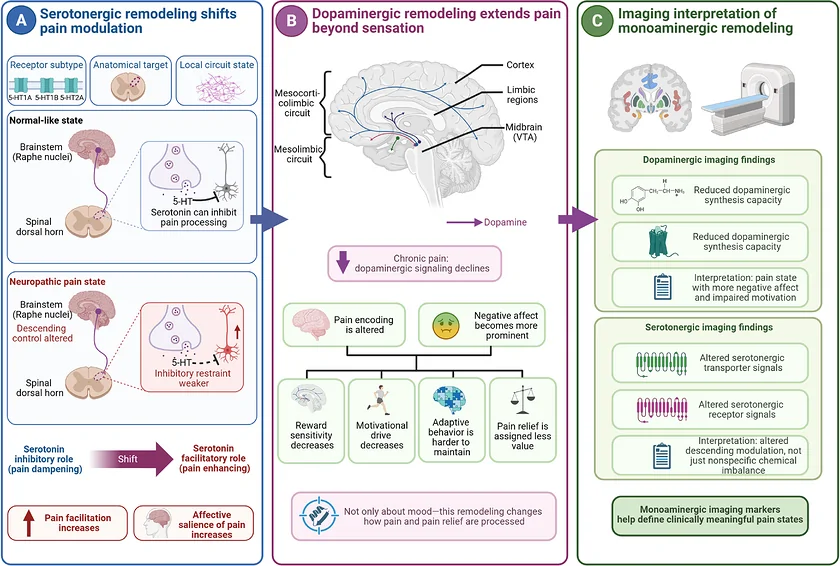
Serotonergic and dopaminergic remodeling links neuropathic pain to affective and motivational dysfunction. (A) The effect of serotonin on pain processing depends on receptor subtype, anatomical target, and local circuit state. In neuropathic pain, impaired descending control and weaker inhibitory restraint may shift serotonergic signaling from an inhibitory to a facilitatory influence. (B) Reduced mesolimbic and mesocorticolimbic dopaminergic signaling alters pain encoding and reward‐related processing, thereby increasing negative affect and impairing motivation and adaptive behavior. (C) Dopaminergic and serotonergic imaging findings should be interpreted as circuit‐level markers of clinically meaningful monoaminergic pain states rather than as nonspecific neurochemical changes. 5‐HT, 5‐hydroxytryptamine (serotonin); 5‐HT1A, serotonin receptor 1A; 5‐HT1B, serotonin receptor 1B; 5‐HT2A, serotonin receptor 2A; VTA, ventral tegmental area. Created with BioRender.com.

### 3.4 Noradrenergic and Neuroimmune Modulation

The role of noradrenergic signaling in neuropathic pain is dynamic, shifting with the state of disease. Under normal conditions, descending noradrenergic projections from the locus coeruleus help suppress nociceptive transmission, partly through spinal α2‐adrenergic mechanisms [64, 65]. In chronic pain states, this may change because descending noradrenergic control itself shows marked plasticity. In some circuits, α2 receptor‐mediated inhibition becomes weaker, while facilitatory adrenergic effects become more evident [27, 64, 65]. In this situation, norepinephrine is no longer only a descending brake; in some chronic pain states, it may become part of an abnormal signaling environment that helps maintain spinal hyperexcitability and central sensitization [27, 64].

This shift is also closely tied to neuroimmune modulation. Once microglia and astrocytes are activated, they can release cytokines, reactive oxygen species, BDNF, and other mediators, which may alter synaptic transmission and reduce inhibitory control [27, 66, 67]. Glial cells, in turn, express noradrenergic receptors, which allow stress‐related or injury‐related noradrenergic signals to act more directly on inflammatory pathways [66]. Under these conditions, adrenergic signaling and neuroinflammation may reinforce one another, rather than acting as separate processes. This interaction may increase excitatory transmission, disturb chloride homeostasis, and keep pain‐related plasticity active for a longer period.

A neuroimmune reading of noradrenergic biology may also make the clinical interpretation more useful. It helps explain a common clinical problem: drugs that raise descending monoaminergic tone can reduce neuropathic pain in some patients, but their effect may be weaker when glial activation and central sensitization have already become clear parts of the pain state [27, 68]. In imaging work, noradrenergic transporter signals and neuroinflammatory PET readouts may therefore be more informative when they are read together, rather than as two unrelated findings [40]. A mechanism‐based model also allows different patient patterns to be separated more carefully. One patient may show a phenotype mainly shaped by monoaminergic dysfunction, while another may show a stronger adrenergic‐glial facilitation pattern. (Figure 5)

**FIGURE 5:**
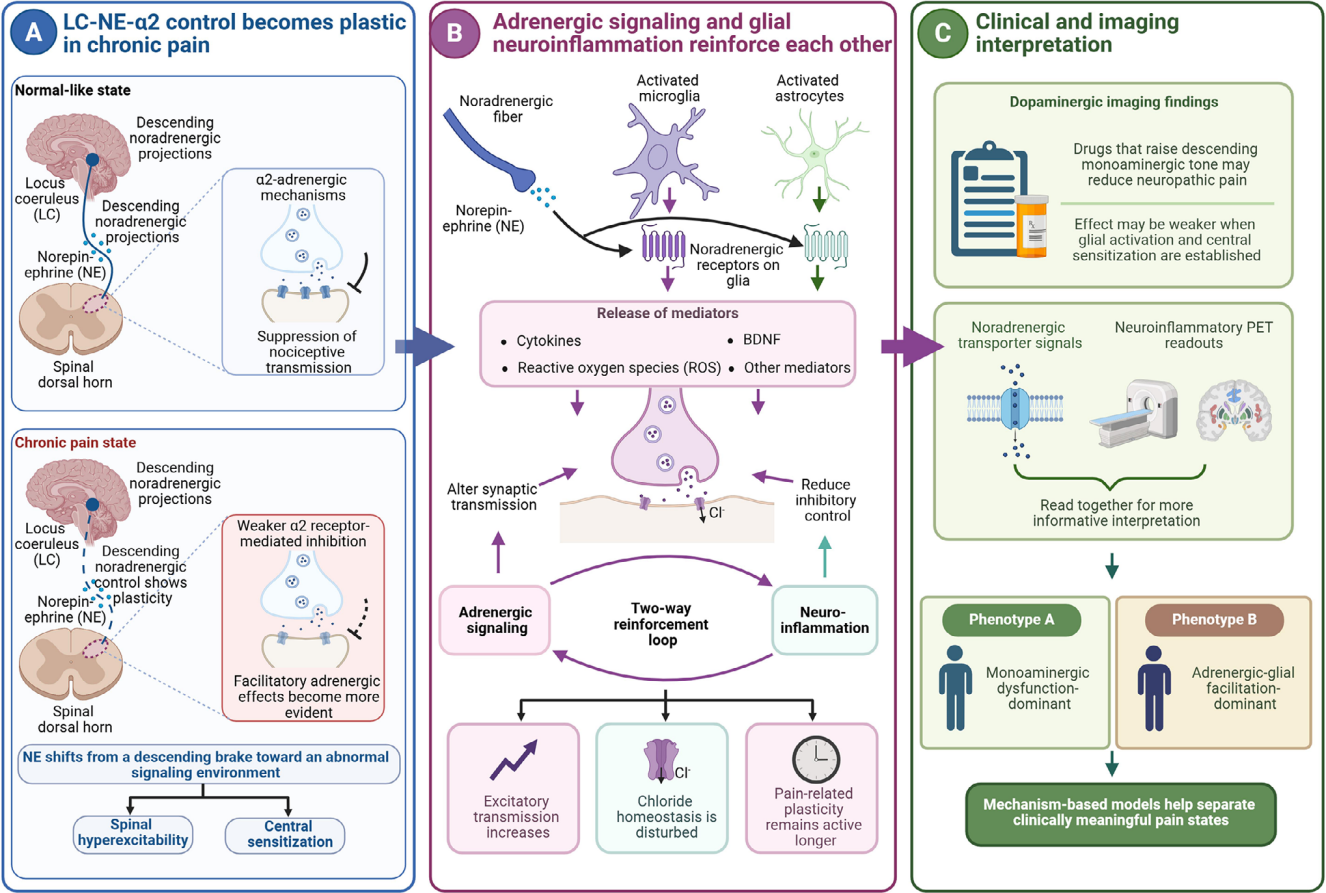
Plasticity of descending noradrenergic control and glial activation reinforces neuropathic pain maintenance. (A) Under normal conditions, descending projections from the LC suppress nociceptive transmission through spinal α2‐adrenergic mechanisms; in chronic pain, reduced α2 receptor‐mediated inhibition and stronger facilitatory adrenergic effects promote spinal hyperexcitability and central sensitization. (B) Noradrenergic signaling interacts with activated microglia and astrocytes, whose release of cytokines, ROS, BDNF, and other mediators alters synaptic transmission, weakens inhibitory control, and disturbs chloride homeostasis. (C) The combined interpretation of noradrenergic transporter signals and neuroinflammatory PET findings may distinguish pain states dominated by monoaminergic dysfunction from those characterized by adrenergic‐glial facilitation. α2, alpha‐2 adrenergic receptor; BDNF, brain‐derived neurotrophic factor; Cl^−^, chloride ion; LC, locus coeruleus; NE, norepinephrine; PET, positron emission tomography; ROS, reactive oxygen species. Created with BioRender.com.

## 4 Neural Circuit Remodeling and Network Reorganization in Neuropathic Pain

Neural circuits linked to pain undergo structural remodeling; only then can underlying molecular defects trigger observable clinical symptoms. For patients living with neuropathic pain, flawed spinal gating mechanisms boost nociceptive signals sent toward higher brain regions. Alongside this shift, thalamo‐cortical circuits, limbic areas, reward networks, and descending regulatory pathways slowly develop harmful activity rhythms and abnormal interconnections. Such widespread reconfiguration across pain networks clarifies a key clinical observation. Many individuals enduring long‐term pain suffer from allodynia, poor emotional wellbeing, loss of drive, and cognitive struggles. These troubles persist regardless of whether the initial peripheral injury remains the main trigger of discomfort.

### 4.1 Spinal and Ascending Amplification Pathways

#### 4.1.1 Spinal Disinhibitory Microcircuits

Recent studies have shown that a subset of dorsal root ganglion neurons are capable of synthesizing gamma‐aminobutyric acid (GABA) and are immunoreactive for the vesicular GABA transporter (Vgat), which packages GABA into synaptic vesicles essential for inhibitory neurotransmission. The central terminals of Vgat‐positive primary sensory neurons form inhibitory connections with spinal somatostatin‐positive excitatory interneurons while also establishing glutamatergic connections with local GABAergic interneurons, thereby driving feed‑forward inhibition. After nerve injury, reduced inhibitory drive from Vgat‐positive primary sensory neurons onto spinal somatostatin‐positive interneurons is associated with mechanical nociceptive hypersensitivity. Optogenetic or chemogenetic stimulation of Vgat‐positive primary sensory neurons effectively alleviates mechanical allodynia induced by nerve injury [69]. The network's spinal root is a set of disinhibitory microcircuits in the superficial dorsal horn [70]. Lamina I Neurokinin 1 receptor‑positive (NK1R^+^) projection neurons become hyperresponsive when lamina II inhibitory gates fail. That failure is multifactorial: Gamma‑aminobutyric acid type A (GABA_A_) receptor composition shifts (α1/α2/δ down, α3 up), Glycine receptor (GlyR) α1/α3 undergoes endocytosis, and KCC2 cleavage drives chloride reversal toward depolarization, converting GABAergic currents from inhibitory to excitatory. Astrocytes downregulate excitatory amino acid transporter 2 (EAAT2), microglia release brain‑derived neurotrophic factor (BDNF), which further depresses KCC2, and D‐serine from astrocytes antagonizes glycinergic signaling [71]. At the same time, protein kinase C gamma‑positive (PKCγ^+^) excitatory interneurons in lamina III and calretinin^+^ amplifiers become persistently active, opening a pathological bridge by which Aβ low‐threshold mechanoreceptor input accesses lamina I projection neurons—an anatomical substrate for tactile allodynia.

#### 4.1.2 Thalamo–Cortical Dysrhythmia and Sensory Amplification

Parallel maladaptation unfolds in thalamo–cortical relays. Abnormal intrinsic low‐frequency oscillations within the thalamocortical network disrupt normal state‑dependent information flow between the thalamus and cortex [72]. Functional magnetic resonance imaging (fMRI) studies have also shown that aberrant low‐frequency oscillations and connectivity in the thalamocortical network underlie persistent pain perception [73]. Within the ventral posterior nuclei (VPL/VPm) and medial dorsal thalamus, relay neurons slip into burst mode as Cav3.2 T‐type currents predominate and GABAergic control from the reticular thalamic nucleus wanes [74, 75]. At the receptor level, thalamic GABA_A_ α1/δ subunits fall while α4/α5 rise; chloride extrusion via KCC2 weakens, shifting inhibitory reversal potentials toward depolarization. Downstream, S1/S2 and posterior insular cortices acquire exaggerated recurrent excitation as parvalbumin‐positive GABAergic (PV^+^) interneuron reliability drops and astrocytic glutamate clearance is impaired.

#### 4.1.3 Parabrachial–Amygdala Aversive Relay

A crucial supraspinal bridge between nociception and affect is the lateral parabrachial nucleus to central amygdala axis [76, 77, 78]. Lamina I spino‐parabrachial projection neurons drive vesicular glutamate transporter 2 (VGLUT2)^+^ cells in the external lateral PBN; chronic injury pushes this hub into a hypermetabolic, Fos‐high state with strengthened vesicular glutamate transport and NK1‐receptor tone [79]. Dense projections to the central amygdala recruit corticotropin‐releasing factor (CRF)^+^ and somatostatin (SOM)^+^ ensembles in the centrolateral amygdala (CeL), remodeling the protein kinase C delta (PKC‐δ^+^)/somatostatin (SOM^+^) microcircuit that gates the centromedial amygdala (CeM) output to brainstem sites [80, 81]. In the central amygdala (CeA), AMPA receptor subunit GluA1 (GluA1) phosphorylation at Serine 831, metabotropic glutamate receptor 5 (mGluR5) accumulation, a shift toward 5‐hydroxytryptamine (5‐HT)_2_A dominance over 5‐HT_1_A/_3_, and a drop in cannabinoid receptor type 1 (CB1) receptor availability reconfigure synaptic gain toward aversion and hypervigilance. Lateral habenula (LHb) engagement—via lateral parabrachial nucleus (Lpbn)–CeA–LHb routes—adds tonic glutamatergic drive to the aversive axis [82, 83].

### 4.2 Affective‐Aversive and Salience Circuits

#### 4.2.1 Anterior Cingulate–Ventral Tegmental Area (ACC–VTA) Loop

The anterior cingulate–ventral tegmental area loop illustrates how cortical appraisal becomes entwined with mesocortical dopamine [63, 84]. Studies have confirmed that a positive feedback loop in the ACC–VTA circuit drives the progression and maintenance of persistent pain, accompanied by anxiety‐like behaviors [84]. In chronic pain, layer V/VI pyramidal neurons in the dorsal ACC (areas 24/32) exhibit a shift toward high‐gain firing driven by GluN2B‐skewed NMDA signaling, CP‐AMPAR insertion, and a loss of M‐current restraint [85]. Astrocytic EAAT2 downregulation and mGluR5 accumulation, including intranuclear membrane pools, further bias calcium signaling and ERK–CREB programs that stabilize excitability and dendritic spine density. These pyramidal ensembles project dense VGLUT1^+^ input to VTA GABA interneurons, strengthening feedforward inhibition of neighboring dopaminergic neurons. Within the VTA, dopamine (DA) cells show lower hyperpolarization‐activated cyclic nucleotide‐gated channel (HCN) and hyperpolarization‐activated cyclic nucleotide‐gated channel (GIRK) currents and heightened GABA_A_ α2‐mediated inhibition; dopamine synthesis and phasic bursting decline, weakening mesocortical feedback to the ACC. Resting‐state fMRI typically reveals stronger ACC–VTA coupling yet reduced flexibility of this linkage, while ligand PET demonstrates reduced striatal D2/3 availability during reward challenges, decreased [^18^F]‐FDOPA uptake along the mesocorticolimbic axis, and increased ACC mGluR5 binding [86]. These fingerprints align with clinical phenomena—catastrophizing, anhedonia, and cognitive perseveration—and offer levers: mGluR5 negative modulators or K^+^ voltage‐gated channel subfamily Q member (KCNQ) openers to tame ACC gain, dopaminergic modulators to restore mesocortical tone, and noninvasive stimulation of dorsal ACC to break the positive‐feedback loop.

#### 4.2.2 Insula‐Centric Salience and Interoception

The insula‐centric salience network provides a complementary interoceptive frame [87, 88]. The anterior insula integrates the visceral and nociceptive context with the autonomic state, and in neuropathic pain, it acquires a persistent high‐metabolic baseline with a raised glutamate‐to‐GABA ratio. PV^+^ interneuron hypofunction and mGluR5 accumulation facilitate pyramidal synchrony; β2‐adrenergic signaling and diminished CB1 receptor tone bias the network toward the oversalience of pain cues [89]. Anterior insula connectivity to the dorsal ACC and opercular somatosensory fields rises, baseline cerebral blood flow increases, and network analyses suggest elevated participation coefficients with reduced modular segregation. Pupillometry and fMRI fluctuations indicate tight coupling of autonomic arousal to anterior insula activity during pain flares [90].

### 4.3 Valuation, Motivation, and Reward/Antireward Coupling

#### 4.3.1 Ventral Hippocampus–LHb–RMTg–VTA Valuation Chain

The valuation arm of the pain connectome is captured by a ventral hippocampus–LHb–rostromedial tegmental nucleus (RMTg)–VTA chain [91]. In chronic pain, ventral hippocampal Cornu Ammonis 1 (CA1)/subiculum pyramidal neurons bias their outputs toward the LHb at the expense of nucleus accumbens pathways [92], and LHb neurons reduce SK‐channel restraint and acquire tonic, high‐gain firing that drives the GABAergic rostromedial tegmental nucleus. The RMTg in turn potently inhibits VTA dopaminergic ensembles, flattening the phasic DA signals necessary for motivation and contextual safety learning [93]. Epigenetic repression of dopamine D1 receptor (D1)/dopamine D2 receptor (D2) receptor transcription in the ventral hippocampus, dendritic spine loss, and NMDA receptor subunit 2B (NR2B) reduction in vHPC targets consolidate this state. Imaging converges on heightened functional coupling among the vHPC, LHb, and VTA; synaptic density appears increased in the LHb; and dopaminergic synthesis markers decline in the midbrain [94]. These signatures align with anhedonia and low motivation, and they nominate habenular deep brain stimulation (DBS), SK‐channel openers, dopaminergic partial agonism, and experience‐based environmental enrichment as feasible interventions [95].

#### 4.3.2 Cerebello–Thalamo–Cortical Prediction Loops

Pain‐related prediction and sensory–motor integration receive a nontrivial contribution from cerebello–thalamo–cortical loops [96, 97]. Purkinje cells in Crus I/II and lobule VI alter simple‐spike statistics, granule‐cell drive rises, and deep nuclear output (particularly dentate) becomes more phasic. Through the superior cerebellar peduncle, cerebellar bursts re‐entrain the ventral lateral thalamic nucleus (VL)/mediodorsal thalamic nucleus (MD) and premotor/prefrontal cortices, amplifying somatosensory prediction errors and salience tagging. Kainate receptor regulation and α2δ‐subunit signaling reshape Purkinje synaptic gain, while endocannabinoid tone declines, diminishing adaptive plasticity. fMRI shows dentate–VL coupling gains and greater recruitment of cerebellar hubs into salience/executive modules [97]; ^18^F‐fluorodeoxyglucose positron emission tomography (FDG PET) often reveals dentate hypermetabolism; tractography identifies altered microstructure within the superior cerebellar peduncle (SCP) [98]. These observations do not imply a single cerebellar “pain center” but rather a recalibration failure across predictive coding loops—an attractive substrate for cerebellar transcranial direct current stimulation (tDCS)/transcranial magnetic stimulation (TMS) and graded motor learning paradigms.

### 4.4 Descending Control Failure and Brainstem Modulation

#### 4.4.1 Amygdala–Prefrontal Cortex–Periaqueductal Gray (Amygdala–PFC–PAG) Descending Control

Descending control falters within the amygdala–prefrontal–midbrain axis [99]. Hyperactive basolateral amygdala outputs preferentially recruit PV^+^ interneurons in the medial prefrontal cortex, suppressing layer V pyramidal drive from prelimbic and infralimbic cortices to the periaqueductal gray [100, 101]. In the PAG, column‐specific arrangements (ventrolateral vs. dorsolateral) that would normally relay excitatory control to the rostral ventromedial medulla become dominated by amygdala bias, tilting the balance of rostral ventromedial medulla (RVM) on‐cells and off‐cells toward facilitation. Serotonergic and noradrenergic release in the dorsal horn declines as a result; within the cord, α2‐adrenoceptor signaling desensitizes while α1/β2 tone rises, transforming noradrenergic influence from inhibitory to facilitatory [65]. Imaging recapitulates these steps: effective connectivity from the mPFC to PAG weakens, amygdala–PAG coupling strengthens, μ‐opioid receptor availability falls in limbic and PAG territories, and transporter maps suggest descending monoaminergic scarcity [102]. This axis underlies the clinical utility of SNRIs and α2‐agonists, motivates μ‐opioid–sparing strategies with targeted augmentation of endogenous opioid tone, and provides a rationale for intermittent theta burst stimulation (iTBS) over the DLPFC/mPFC to reinvigorate cortical control [103, 104].

#### 4.4.2 Monoaminergic Brainstem Hubs and Chemical Climate of Control

Monoaminergic brainstem hubs define the chemical climate of descending control [105, 106]. In neuropathic pain models, the activity of excitatory serotonin 3 (5‐HT3) receptors is exacerbated. Opioids are involved in modulating descending modulatory circuits. During neuropathic pain, opioid modulation in brainstem pain control regions undergoes changes, characterized by enhanced local opioid release, along with reduced expression and desensitization of mu‐opioid receptors (MORs) [107]. Dorsal raphe serotonergic projections to the amygdala, ACC, and PAG undergo a receptor‐level switch in targets: 5‐HT_2_A dominance in amygdala projection neurons replaces 5‐HT_1_A/_3_‐mediated inhibition, transforming serotonin from a brake into a facilitator of aversive output. Locus coeruleus neurons increase tonic firing; at the cord, α2‐adrenoceptors desensitize, while α1/β2 signaling strengthens, aided by astrocytic β2‐adrenoceptor–driven cytokine release that accelerates glutamate vesicle mobilization. Microglial BDNF reduces KCC2, making GABAergic currents less inhibitory. Imaging with transporter ligands shows lower SERT and NET binding along descending pathways, while brainstem‐resolved fMRI identifies altered PAG–RVM coupling [108]. Clinically, these data explain the efficacy of SNRIs and α2‐agonists, argue for receptor‐subtype–selective serotonergic strategies, and highlight aerobic exercise as a circuit‐wide normalizer of LC phasic/tonic balance.

### 4.5 Large‐Scale Network Reconfiguration

#### 4.5.1 Default Mode and Executive Control Reconfiguration

Large‐scale network reconfiguration extends into the default mode and executive control systems [109, 110]. Posterior cingulate/precuneus and medial prefrontal nodes in the default mode network show blunted task‐induced deactivation and persistent low‐frequency synchrony, while dorsolateral prefrontal executive nodes lose anticorrelation with both salience and sensorimotor networks. Microstructurally, the cingulum bundle and callosal midbody show reduced anisotropy; biochemically, cortical GABA tone falls, while astrocytic glutamate handling is compromised [111, 112]. Successful analgesia—pharmacologic or device‐based—often restores DMN–ECN segregation on fMRI, supporting DLPFC‐directed iTBS and metacognitive training that enhances attentional set‐shifting as network‐level treatments.

#### 4.5.2 Synthesis and Translational Levels

Taken together, these circuits compose a set of repeating motifs. Ascending amplification arises from thalamic and parabrachial glutamatergic surplus layered onto cortical disinhibition; descending failure reflects weakened mPFC→PAG influence and monoaminergic transporter loss; valuation coupling is enforced by vHPC–LHb–VTA and AI–ACC signatures that bind nociception to negative affect and cognitive load; and spinal disinhibition ensures low‐threshold input access projection neurons [70, 91, 113]. Multimodal imaging permits each motif to be measured: ligand PET for receptor/transporter availability and neuroinflammation, spectroscopy for local E/I balance, tractography for pathway integrity, and fMRI/MEG for effective connectivity and oscillatory state [114, 115]. This same measurement suite provides handles for precision therapeutics: mGluR5 modulators for thalamic/insula excess, T‐type channel agents and thalamic DBS for dysrhythmia, CeA/PBN neuromodulation for aversive gain, α2‐agonists and SNRIs for descending repair, KCC2 and CP‐AMPAR strategies for spinal gating, habenular or cerebellar stimulation for valuation and prediction coding, and DLPFC/mPFC stimulation with cognitive training to restore network segregation [116, 117, 118, 119, 120, 121, 122]. The critical point is not that one circuit explains all pain but that each patient expresses a dominant motif—or a small combination—that can be phenotyped by imaging and targeted by mechanism‐aligned interventions, with the same imaging markers closing the loop as objective outcome measures (Table 1).

**TABLE 1:** Molecular and circuit mechanisms linked to imaging readouts in neuropathic pain.

Mechanism or circuit	Main biological change	Main regions or pathways	Main imaging readout	Mechanistic implication
Glutamatergic dysregulation	Astrocytic glutamate uptake is reduced. GluN2B‐NMDA signaling, GluA1 insertion, calcium‐permeable AMPA receptors, and mGluR5 signaling increase synaptic gain	Spinal dorsal horn, thalamus, insula, anterior cingulate cortex, and hippocampus	MRS may show altered glutamate‐GABA balance. PET may show mGluR5‐related changes. fMRI may show stronger thalamo‐cortical or limbic coupling	This pattern indicates excitatory gain and central sensitization [27, 40, 50, 56, 57]
GABAergic disinhibition	GABAergic inhibitory control weakens because inhibitory synapses, GABAA receptor composition, and inhibitory interneuron function are altered	Spinal dorsal horn and supraspinal inhibitory networks	MRS may show reduced inhibitory metabolites. PET may show reduced GABAA‐related binding	This pattern indicates loss of inhibitory control and greater vulnerability to spontaneous or evoked pain [27, 40, 58, 59]
Glycinergic disinhibition	GlyT2 activity decreases, glycinergic inhibition falls, and microglia‐derived BDNF may reduce KCC2 function	Spinal dorsal horn inhibitory circuits	MRS and other inhibitory readouts may indirectly reflect reduced inhibitory tone	This pattern indicates dorsal horn disinhibition and easier activation of nociceptive circuits by weak sensory input [27, 58, 59]
Serotonergic remodeling	Serotonin signaling may shift from inhibition toward facilitation in some pain circuits. This shift depends on receptor subtype, anatomical target, and circuit state	Descending pathways, amygdala, ACC, PAG, and spinal dorsal horn	PET may show altered SERT or receptor‐related signals. fMRI may show altered descending modulation	This pattern suggests that descending control may no longer act mainly as a brake on pain [27, 40, 60, 61, 62]
Dopaminergic remodeling	Mesolimbic and mesocorticolimbic dopamine signaling declines. reward, motivation, and pain valuation may be affected	VTA, ventral hippocampus, nucleus accumbens, prefrontal cortex, and limbic circuits	PET may show reduced dopamine synthesis or lower D2/3 receptor availability. fMRI may show altered reward‐network connectivity	This pattern links chronic pain with negative affect, impaired motivation, and reduced adaptive behavior [27, 40, 63]
Noradrenergic and neuroimmune modulation	Descending noradrenergic control may shift from alpha2‐mediated inhibition toward facilitation. Activated glia release cytokines, reactive oxygen species, and BDNF	Locus coeruleus, spinal dorsal horn, astrocytes, microglia, and descending pathways	NET PET and neuroinflammatory PET may help identify adrenergic‐glial patterns	This pattern suggests that monoaminergic dysfunction and glial activation may reinforce each other [27, 40, 64, 65, 66, 67, 68]
Spinal disinhibitory microcircuits	Lamina II inhibitory gates fail, and lamina I projection neurons become more responsive. PKCgamma‐positive and calretinin‐related circuits allow low‐threshold input to activate pain pathways	Superficial dorsal horn, lamina I projection neurons, lamina II inhibitory circuits, and A‐beta input pathways	Spinal fMRI may show increased responses to innocuous stimulation. MRS may show reduced inhibitory tone	This pattern supports tactile allodynia and spinal amplification [69, 70, 71]
Thalamo‐cortical dysrhythmia	Thalamic relay neurons show abnormal low‐frequency oscillations and burst firing. Thalamo‐cortical information flow becomes unstable	VPL, VPm, medial dorsal thalamus, S1, S2, and posterior insula	fMRI, EEG, and MEG may show abnormal oscillations and connectivity. DTI may show altered thalamo‐cortical pathways	This pattern indicates sensory amplification and unstable thalamo‐cortical processing [72, 73, 74, 75]
Parabrachial‐amygdala aversive relay	Spinal input to the lateral parabrachial nucleus and central amygdala strengthens. This pathway links nociception with aversion and hypervigilance	Lateral parabrachial nucleus, central amygdala, and lateral habenula‐related routes	fMRI may show stronger PBN‐CeA coupling. FDG‐PET may show pathway hypermetabolism	This pattern indicates affective amplification of pain [76, 77, 78, 79, 80, 81, 82, 83]

Abbreviations: ACC, anterior cingulate cortex; AMPA, α‐amino‐3‐hydroxy‐5‐methyl‐4‐isoxazolepropionic acid; BDNF, brain‐derived neurotrophic factor; D2/3, dopamine receptor D2/3; fMRI, functional magnetic resonance imaging; GABA, γ‐aminobutyric acid; GABAA receptor, γ‐aminobutyric acid type A receptor; GluA1, glutamate ionotropic receptor AMPA type subunit 1; GluN2B, glutamate ionotropic receptor NMDA type subunit 2B; GlyT2, glycine transporter 2; KCC2, potassium‐chloride cotransporter 2; mGluR5, metabotropic glutamate receptor 5; MRS, magnetic resonance spectroscopy; NET, norepinephrine transporter; NMDA, N‐methyl‐D‐aspartate; PAG, periaqueductal gray; PET, positron emission tomography; SERT, serotonin transporter; VTA, ventral tegmental area.

## 5 Peripheral and Central Imaging Approaches

Clinicians cannot reliably pinpoint where pain sustains itself anatomically or determine the relevant biological mechanisms merely based on symptoms and conventional neurophysiological examinations. Imaging targeted at peripheral nerves enables detection of structural damage, nerve entrapment, and mechanical nerve anomalies. In contrast, brain and spinal imaging tools uncover molecular shifts, metabolic fluctuations, tissue remodeling, and large‐scale network rearrangements linked to persistent pain. Combining these imaging modalities creates opportunities to map each patient's clinical manifestations to peripheral and central factors that sustain pain. This strategy avoids the oversimplified practice of categorizing all neuropathic pain cases under one unified diagnostic framework.

### 5.1 Peripheral Nerve Ultrasound and MR Neurography

Peripheral nerve ultrasound and MR neurography should be used as complementary techniques rather than competitive alternatives. Ultrasound can be utilized bedside within the clinic setting, is low cost, and allows for the observation of nerve dynamics with movement [38, 123, 124]. Ultrasound can identify nerve enlargement and caliber changes, distortion and loss of nerve fascicular pattern, changes in echogenicity, hypervascularity, reduced nerve excursion, and secondary muscle denervation and atrophy. In clinical settings, ultrasound is preferentially used for cases with suspected focal entrapment, injury to superficial nerves, postsurgical changes, and dynamic movements between nerve and surrounding tissues [38, 123]. MR neurography provides extensive coverage of anatomy, visualization of deep anatomic nerves and plexuses, depiction of intraneural edema and abnormal signal, scarring, and surrounding alterations in the soft tissue [38, 123, 124, 125]. MR and ultrasound together provide a more comprehensive perspective of the site of damage, in relation to the surrounding tissue, and the extent of the structural changes compared with the narrative of physical symptoms [123, 124, 125].

In addition, peripheral nerve ultrasound can be considered for inclusion during the stratification process. The presence of focal nerve enlargement, changes in nerve caliber, distortion of nerve fascicles, changes in nerve echogenicity, hypervascularity, and dynamic nerve compression at sites of potential entrapment can aid in the diagnosis of a putative structural peripheral pain generator [38, 123, 126, 127]. This is especially the case when abnormal nerve imaging findings correlate with the anatomical territory of sensation and the neurological examination, as this links the observed nerve imaging abnormality with the clinical pattern of the patient. Clinicians should not exclude neuropathic pain because nerve ultrasound findings are normal. They should interpret imaging alongside the clinical history and neurological examination. Further testing should address the suspected nerve disorder [38].

Peripheral nerve ultrasound can also be used to correlate nerve morphology with potential mechanisms of pain. Nerve enlargement, increased nerve fascicular size, or altered nerve fascicular morphology may be suggestive of nerve compression, edema, inflammatory changes, or chronic structural changes of the nerve [38, 123, 126, 127]. Dynamic ultrasound can further identify whether nerve structures may be compressed, displaced, or irritated with movements that mechanically provoke pain symptoms, such as in entrapment neuropathies [127]. These findings help identify peripheral nerve abnormalities. Clinicians should assess whether the imaging findings match the distribution of symptoms and the neurological examination before selecting a local treatment [38, 123, 127, 128].

These imaging methods can also help with clinical grouping, especially when the pain source is not clear from symptoms alone. When ultrasound or MR neurography shows nerve enlargement, abnormal fascicular structure, focal edema, or entrapment‐related distortion, the pain condition is more likely to be linked to a structural peripheral lesion [38, 123, 124, 125]. The opposite situation is also important. If neuropathic symptoms are present but morphologic changes are absent or only mild, clinicians need to consider other explanations, such as small‐fiber‐predominant disease or a pain state in which central mechanisms have become more involved [50, 129]. In this sense, imaging is not only used to show whether a lesion is present; it may also help indicate which biological level is mainly maintaining the current pain state [38, 123, 124, 125].

Newer nerve imaging methods may also extend this role. One example is ultrasound elastography, which evaluates the mechanical properties of nerves rather than the morphology alone [129, 130]. Changes in nerve stiffness and morphology have been correlated with peripheral neuropathic involvement in type 2 diabetes [129, 130]. These techniques are promising and under continuous development, as they allow the connection of nerve‐specific pathology with pain phenotypes [38, 129]. Longitudinal peripheral imaging may also help record changes in nerve morphology and nerve mechanics during treatment; however, this application still needs stronger clinical evidence [38, 123, 129].

### 5.2 Central Molecular and Network Imaging

Central imaging can be particularly valuable when ongoing pain cannot be fully explained by peripheral pathology. PET reveals receptor availability, transporter signals, synaptic targets, and neuroinflammation [40]. Metabolites thought to reflect the local balance between excitatory and inhibitory neurochemicals can be quantified using MRS [131]. Structural and diffusion MRI can reveal local volume loss and impaired structural integrity of white matter tracts, while functional MRI may reveal aberrant connectivity patterns in thalamo‐cortical, salience, limbic, and inhibitory descending pathways [131, 132].

Central imaging should not be used solely to describe changes in the brain. In neuropathic pain, central imaging may provide a method to bridge the observed clinical phenotype with the mechanism, albeit indirectly. For patients in whom pain is more dependent on sensory input, the abnormalities may be most apparent along the thalamo‐somatosensory route, whereas for patients with a greater affective burden, changes in the insula, cingulate cortex, or other cortico‐limbic connections may be more relevant [39, 133]. PET findings with respect to glutamatergic, GABAergic, or neuroinflammatory signaling may provide additional clues about why individuals with similar symptom profiles may differ in their response to treatment [40, 131]. Hence, the utility of central imaging does not just reside in its ability to document brain changes associated with chronic pain; it provides a way to contextualize those changes within the phenotype‐mechanism framework.

Brain MRI studies have examined treatment response and sensory profiles in painful diabetic neuropathy. One study found differences in brain structure and functional connectivity between responders and nonresponders to intravenous lidocaine [39]. Another study found differences in resting‐state functional connectivity between sensory phenotypes [133]. Future studies should test whether changes in these measures track treatment response over time.

A practical next step would be to assess peripheral and central imaging in the same patient during the treatment course [38, 39]. For example, in patients with entrapment‐related or mixed neuropathic pain, improvement after a targeted intervention could be examined together with changes at both levels. Peripheral imaging may show whether the local nerve abnormality has improved, while central imaging may show whether this change is accompanied by altered connectivity or molecular signals. Such paired measurements may help clarify how peripheral generators, central sensitization, and clinical outcome are related to each other. Imaging may also gain more value in clinical practice if it can indicate not only the baseline pain category but also the main biological driver at a particular stage of treatment (Table 2) [38, 39, 40].

**TABLE 2:** Clinical stratification of neuropathic pain and matched imaging approaches.

Clinical dimension	Main clinical features	Main mechanism	Useful assessment or imaging method	Main value
Sensory gain phenotype	Patients may report burning pain, electric shock‐like pain, shooting pain, mechanical allodynia, or thermal hyperalgesia	Retained but sensitized nociceptive pathways may show ectopic firing, disinhibition, and central sensitization	Bedside sensory examination, quantitative sensory testing, pain drawings, and central imaging when central sensitization is suspected	This phenotype helps identify patients with active sensory amplification. It may also explain different responses among patients with the same disease diagnosis [24, 25, 26, 27, 28, 29, 30, 31, 32, 33]
Sensory loss phenotype	Patients may show numbness and reduced heat, cold, or touch sensation	Deafferentation and loss of peripheral sensory input may dominate. Central amplification may also occur in some patients	Bedside sensory examination, quantitative sensory testing, nerve conduction studies, and skin biopsy when small‐fiber disease is suspected	This phenotype helps separate sensory loss‐dominant patients from patients with mainly sensory gain [24, 25, 28, 29, 30, 31, 32, 33]
Peripheral pain generator	Pain is linked to a peripheral nerve, dorsal root ganglion, nerve root, or plexus. Typical examples include diabetic neuropathy, postherpetic neuralgia, postsurgical nerve injury, and entrapment neuropathy	Axonal injury, abnormal membrane excitability, ectopic discharge, ion‐channel remodeling, and local neuroimmune changes may maintain pain	Peripheral nerve ultrasound, MR neurography, electrodiagnostic testing, and quantitative sensory testing	This category helps locate a structural peripheral pain source and clarify whether local nerve pathology is the main driver [27, 34, 35, 36, 37, 38, 39, 40, 123, 124, 125, 126, 127, 128]
Central pain generator	Pain follows a lesion or disease in the brain or spinal cord, such as poststroke pain, spinal cord injury pain, or multiple sclerosis‐related pain	Loss of sensory input, abnormal synaptic plasticity, reduced descending inhibition, and central network remodeling may maintain pain	PET, MRS, structural MRI, DTI, fMRI, and network analysis	This category helps explain persistent pain when peripheral findings are absent, mild, or not matched with symptom severity [27, 34, 35, 36, 37, 38, 39, 40, 131, 132, 133]
Mixed nociceptive‐neuropathic pain	Neuropathic symptoms coexist with musculoskeletal pain, radicular pain, postsurgical pain, or entrapment‐related pain	Nerve injury and nociceptive tissue load may amplify each other. Mechanical stress, local inflammation, and impaired nerve gliding may contribute	Musculoskeletal examination, ultrasound, MR neurography, sensory testing, and central imaging when pain is widespread or disproportionate	This category helps avoid treating complex pain as purely neuropathic or purely nociceptive [41, 42, 43, 44, 45, 46, 47]
Central sensitization modifier	Patients may show temporal summation, widespread pain, allodynia, and a mismatch between lesion size and symptom burden	Persistent amplification in spinal and supraspinal circuits may maintain pain beyond the original lesion	Quantitative sensory testing, fMRI, MRS, PET, and connectivity analysis	This modifier helps identify patients whose pain is strongly maintained by central amplification [27, 48, 51, 52]
Autonomic and small‐fiber modifier	Patients may show burning pain, dysesthesia, heat intolerance, vasomotor symptoms, gastrointestinal symptoms, sweating changes, or orthostatic intolerance	Small‐fiber involvement and altered autonomic regulation may contribute to symptom fluctuation and pain severity	Autonomic testing, skin biopsy, quantitative sensory testing, and clinical autonomic assessment	This modifier helps explain symptoms that cannot be fully explained by lesion anatomy alone [49, 50, 53, 54, 55]
Peripheral nerve ultrasound	Ultrasound may show nerve enlargement, fascicular distortion, altered echogenicity, hypervascularity, reduced nerve movement, or dynamic compression	Structural nerve injury, compression, edema, inflammation, or mechanical irritation may be present	High‐resolution ultrasound, dynamic ultrasound, and ultrasound elastography	Ultrasound helps identify superficial nerve lesions and dynamic entrapment. It can also support follow‐up of local nerve changes [38, 123, 126, 127, 128, 129, 130]
MR neurography	MR neurography may show deep nerve or plexus lesions, intraneural edema, abnormal signal, scarring, or soft‐tissue changes around nerves	Deep structural nerve injury, plexus involvement, or postinflammatory change may contribute to pain	MR neurography and related fat‐suppressed nerve sequences	MR neurography helps assess deep nerves and broader anatomical regions that are difficult to evaluate with ultrasound alone [38, 123, 124, 125]
Central molecular and network imaging	Central imaging may show abnormal receptor availability, transporter signals, neuroinflammation, white‐matter integrity, and functional connectivity	Central sensitization, transmitter imbalance, glial activation, and network remodeling may maintain pain	PET, MRS, structural MRI, DTI, fMRI, EEG, and multimodal imaging	Central imaging helps connect clinical phenotypes with central mechanisms and may support mechanism‐based stratification [39, 40, 131, 132, 133]

Abbreviations: DTI, diffusion tensor imaging; fMRI, functional magnetic resonance imaging; MR, magnetic resonance; MRI, magnetic resonance imaging; MRS, magnetic resonance spectroscopy; PET, positron emission tomography.

## 6 From Imaging Biomarkers to Therapeutic Interventions

Previously, we elucidated how neuropathic pain remodels the brain's connectome—encompassing limbic and brainstem descending modulatory circuits, cerebellar predictive loops, and large‐scale cortical networks. These maladaptive alterations impart characteristic signatures on structural, functional, and molecular neuroimaging, manifesting as perturbed resting‐state connectivity, diminished fractional anisotropy within white matter tracts, altered receptor availability (e.g., μ‐opioid, dopaminergic, and serotonergic transporters), and metabolic aberrations such as dentate hypermetabolism observed on FDG‑PET. Collectively, these imaging features function not merely as diagnostic correlates but rather as biomarkers that faithfully reflect discrete circuit‐level dysfunctions. These imaging features should not be viewed only as diagnostic correlates. They can also be linked to pathway‐specific interventions. In the following sections, we summarize mechanism‐guided treatment selection, preclinical animal evidence, and recent clinical trials by pathway and mechanism of action (Figure 6).

**FIGURE 6:**
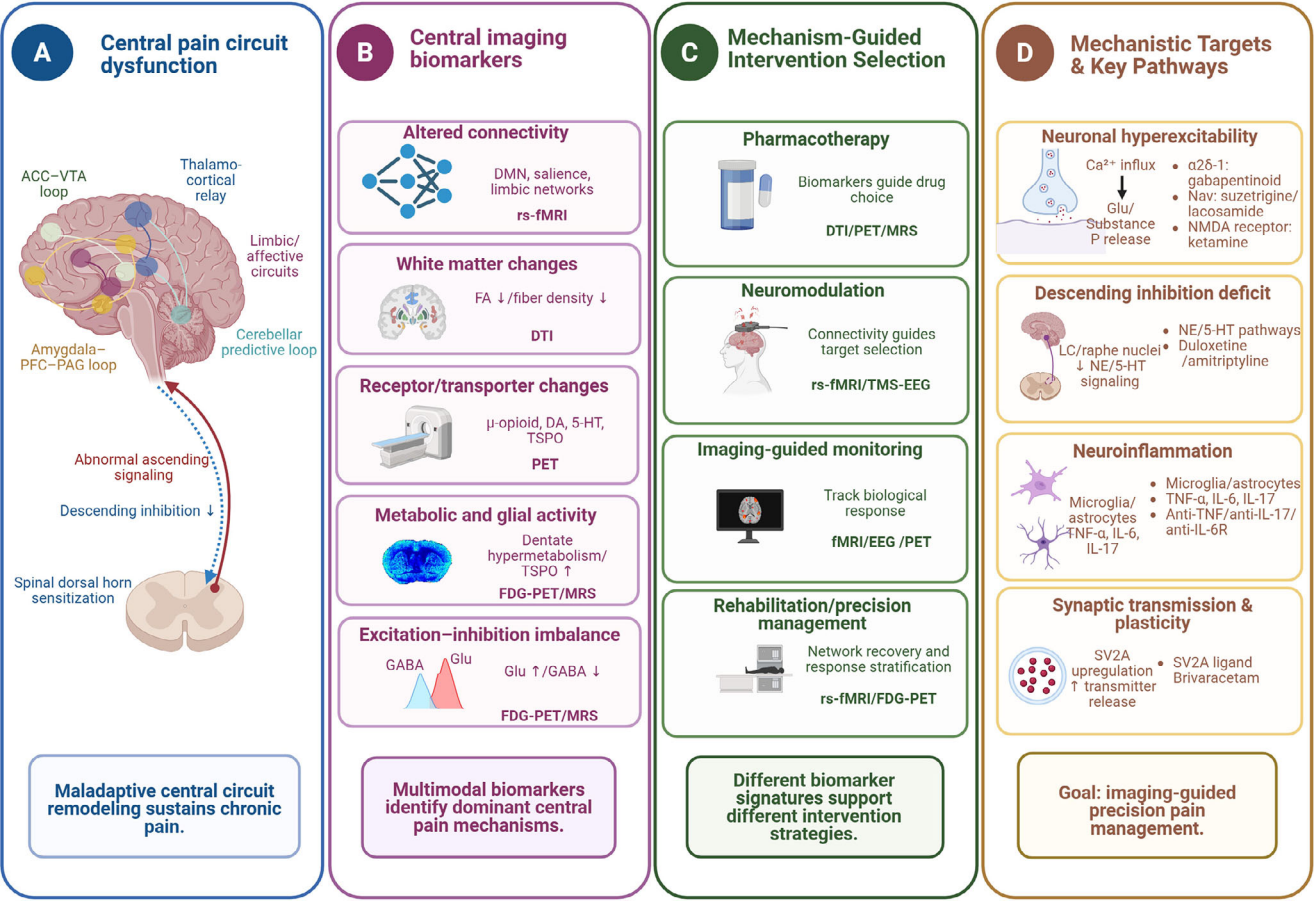
Central imaging biomarkers support mechanism‐guided intervention selection in neuropathic pain. (A) Maladaptive remodeling of thalamocortical, limbic‐affective, ACC–VTA, amygdala–PFC–PAG, cerebellar, and spinal circuits contributes to the persistence of neuropathic pain. (B) These disturbances may be reflected by altered network connectivity, white‐matter microstructure, receptor or transporter signals, metabolic and glial activity, and excitation–inhibition imbalance. (C) Distinct biomarker signatures can inform the selection and monitoring of pharmacotherapy, neuromodulation, imaging‐guided treatment, and rehabilitation‐based management. (D) Mechanistic interventions may target neuronal hyperexcitability, impaired descending inhibition, neuroinflammation, or abnormal synaptic transmission and plasticity. ACC, anterior cingulate cortex; α2δ‐1, alpha‐2‐delta‐1 auxiliary subunit of voltage‐gated calcium channels; DA, dopamine; DMN, default mode network; DTI, diffusion tensor imaging; EEG, electroencephalography; FA, fractional anisotropy; FDG‐PET, fluorodeoxyglucose positron emission tomography; fMRI, functional magnetic resonance imaging; GABA, γ‐aminobutyric acid; Glu, glutamate; IL‐6, interleukin‐6; IL‐17, interleukin‐17; LC, locus coeruleus; MRS, magnetic resonance spectroscopy; NE, norepinephrine; NMDAR, N‐methyl‐D‐aspartate receptor; PAG, periaqueductal gray; PET, positron emission tomography; PFC, prefrontal cortex; rs‐fMRI, resting‐state functional magnetic resonance imaging; SV2A, synaptic vesicle glycoprotein 2A; TMS‐EEG, transcranial magnetic stimulation combined with electroencephalography; TNF‐α, tumor necrosis factor alpha; TSPO, translocator protein; VTA, ventral tegmental area; 5‐HT, 5‐hydroxytryptamine. Created with BioRender.com.

### 6.1 Mechanism‐Guided Treatment Selection

This section examines whether imaging measures can help guide treatment selection and reviews the mechanisms of the treatments discussed.

#### 6.1.1 Guiding Individualized Treatment Selection

Imaging measures may help researchers study treatment response. In patients with neuropathic pain, pretreatment fiber density in the anterior limb of the internal capsule was associated with pain relief after ketamine treatment. Fiber density in the default mode network and the medial prefrontal cortex–periaqueductal gray pathway was associated with changes in temporal summation of pain. These pathway measures did not distinguish responders from nonresponders [134]. In rats with spinal nerve ligation, gabapentin changed activity in limbic and sensorimotor regions and reduced functional connectivity. These effects were greater in injured rats than in sham‐operated rats [135, 136].

A study of 29 patients with chronic intractable pain examined resting‐state fMRI before a spinal cord stimulation trial. Lower functional connectivity between the middle anterior cingulate cortex and the precuneus/posterior cingulate cortex was associated with greater pain relief during the trial [137]. Another study examined 43 patients with chronic pain who received rTMS over the primary motor cortex. Responders had lower pretreatment cortical reactivity and lower alpha‐band responses measured with TMS–EEG. These measures were associated with greater pain relief. The findings require validation in independent patient groups [138].

#### 6.1.2 Mechanism‐Based Pharmacotherapy Selection

Gabapentin and pregabalin bind to α2δ‐1 and reduce excessive excitatory signaling in the spinal dorsal horn. Experimental studies link this effect to the regulation of α2δ‐1‐associated NMDA and AMPA receptors [139, 140].

Voltage‐gated sodium channels (Nav1.7, Nav1.8, Nav1.9) are highly expressed in peripheral nociceptive neurons. They drive abnormal firing in the dorsal root ganglia and primary afferent fibers under disease conditions. Suzetrigine selectively inhibits NaV1.8. Two phase III trials showed that it reduced acute pain after abdominoplasty or bunionectomy compared with placebo. These trials did not assess chronic neuropathic pain [141]. Lacosamide promotes slow inactivation of voltage‐gated sodium channels [142]. In a randomized trial in healthy volunteers, lacosamide reduced the strength‐duration time constant of large sensory fibers. This finding shows a drug effect on peripheral nerve excitability [143]. Duloxetine and amitriptyline work by blocking the reuptake of norepinephrine and serotonin in the synaptic cleft. This strengthens the descending inhibition from the brainstem (like the locus coeruleus and raphe nuclei) down to the spinal dorsal horn, which helps shut the pain gate [144]. Antidepressants also have anti‐inflammatory and pain‐relieving effects through other mechanisms, such as reducing TNF‐α levels in the brain and shifting α2‐adrenoceptor function [145].

Overactivation of NMDA receptors in the spinal dorsal horn can lead to symptoms of neuropathic pain. Ketamine, a noncompetitive NMDA receptor antagonist, can significantly relieve neuropathic pain and central sensitization‐related symptoms [146]. A pilot randomized trial evaluated brivaracetam in patients with spinal cord injury‐related neuropathic pain. The study enrolled 24 participants and included 14 in the final analysis. The results supported further evaluation of the drug in larger trials [147]. Neuroinflammation is a key driver of neuropathic pain. Activated microglia and astrocytes release proinflammatory cytokines such as TNF‐α and IL‐6, which further amplify neuronal hyperexcitability. Experimental studies support neuroimmune pathways as potential treatment targets. These findings do not establish the clinical efficacy of specific cytokine inhibitors for neuropathic pain [148].

### 6.2 Preclinical and Clinical Translational Evidence by Pathway

We distinguish established or guideline‐supported therapies, clinically investigational approaches, and preclinical or mechanistically plausible targets. Gabapentinoids are guideline‐supported first‐line treatments for neuropathic pain [149]. LX9211 has preliminary clinical evidence from a proof‐of‐concept trial [150]. Targeted inhibition of astrocytic mGluR5 has shown benefit in male mice, but this finding does not establish efficacy in patients [151]. Animal studies help researchers test causal mechanisms, including ion‐channel hyperexcitability, spinal disinhibition, altered synaptic vesicle release, neuroimmune activation, and abnormal network activity. Clinical trials are still needed because patients with neuropathic pain differ in disease cause, pain duration, sensory profile, mood symptoms, sleep disturbance, and prior treatment exposure [25, 152, 153, 154].

Researchers should not overlook glial‐related mechanisms within pre‐experimental pain animal models. In mice developing neuropathic pain, stimulating the descending noradrenergic locus coeruleus‐spinal pathway inhibits spinal dorsal horn microglia and astrocyte reactivity. Such regulation promotes a shift toward anti‐inflammatory cytokine patterns and elevates pain thresholds [10]. Supraspinally, work based on chronic constriction injury models shows that astrocytes in the anterior cingulate cortex re‐express mGluR5. This process triggers irregular calcium dynamics, glutamate buildup outside cells, and strengthened excitatory signaling. Targeted suppression of mGluR5 in these astrocytes rescues such neuronal dysfunction and mitigates mechanical allodynia in male mice [151]. These results illustrate the importance of bidirectional glia‐neuron crosstalk in experimental neuropathic pain. Nevertheless, region‐dependent and cell‐type‐specific glial reactions hinder straightforward translation of these laboratory discoveries to patient treatment.

Important translational evidence supports peripheral ion channel modulation and α2δ‐1 calcium channel modulation [149, [152]. Nav1.8 inhibition is a newer approach that aims to reduce abnormal firing in peripheral nociceptors and dorsal root ganglion neurons without direct opioid activity in the central nervous system [155, 156]. α2δ‐1 ligands reduce presynaptic calcium entry and excitatory transmitter release, and these drugs remain a clear example of mechanism‐based treatment for neuropathic pain [157, 158, 159]. Other pharmacological approaches are still at earlier translational stages. These approaches include SV2A modulation, NMDA‐related therapy, AAK1 inhibition, SSTR4 activation, P2X3 antagonism, and cytokine or chemokine targeting [147, 150, 160, 161, 162, 163].

Neuromodulation and focused ultrasound provide another treatment route. These methods do not act on a single receptor. Instead, they aim to change pain‐related circuits [148, 149, 164]. Spinal cord stimulation, rTMS, tDCS, temporal interference stimulation, deep brain stimulation, and focused ultrasound may regulate ascending pain transmission, thalamo‐cortical processing, affective pain circuits, or descending inhibitory control. Imaging is useful in this field because MRI, fMRI, DTI, PET, MRS, EEG, and ultrasound can help define the target, predict treatment response, and monitor biological effects [165, 166, 167]. These techniques differ in their clinical evidence. The 2025 NeuPSIG recommendations give rTMS a weak recommendation as a third‐line treatment for neuropathic pain [149]. Evidence for tDCS remains inconclusive, and focused ultrasound requires further controlled clinical studies [148, 164].

The available evidence also shows important gaps. Many animal studies show clear analgesic effects, but human trials often show mixed results [149, 168]. This mismatch may result from species differences, short observation periods, incomplete phenotyping, and the use of broad clinical diagnoses rather than mechanism‐defined patient groups [25, 153, 168]. Future studies should combine sensory phenotyping, molecular or network imaging, and trial designs based on the main pathway being tested [165, 167]. Current preclinical evidence and recent or ongoing clinical trials are summarized in Tables 3 and 4.

**TABLE 3:** Preclinical animal evidence by pathway and mechanism of action.

Pathway	Experimental model	Intervention or target	Mechanism of action	Main preclinical finding	Translational implication and references
Ion‐channel hyperexcitability	Peripheral nerve injury, diabetic neuropathy, and DRG hyperexcitability models	Nav1.7, Nav1.8, and Nav1.9 modulation	Reduces abnormal firing in nociceptors and DRG neurons	Sodium‐channel modulation reduces ectopic firing and pain‐like behavior in animal models	Supports peripheral ion‐channel blockade as a mechanism‐based strategy for neuropathic pain [27, 155].
α2δ‐1 calcium‐channel pathway	Peripheral nerve injury and spinal sensitization models	Gabapentin, pregabalin, and related α2δ‐1 ligands	Reduces presynaptic calcium entry and excitatory transmitter release	α2δ‐1 ligands reduce allodynia and hyperalgesia in neuropathic pain models	Supports the clinical use of α2δ‐1 ligands in selected patients with neuropathic pain [152, 157, 158, 159]
Glutamate and NMDA‐related plasticity	Nerve injury, chemotherapy‐induced neuropathy, and central sensitization models	NMDA receptor antagonists and NMDA‐related modulators	Reduces glutamate‐driven central sensitization and synaptic gain	NMDA‐related interventions reduce pain‐like behavior in several animal models	Supports clinical testing of NMDA‐related therapies, although tolerability remains important [27, 160]
Spinal disinhibition	Spinal dorsal horn disinhibition after nerve injury	KCC2 restoration and inhibitory circuit activation	Restores inhibitory tone and reduces abnormal low‐threshold input to pain projection neurons	Restoration of inhibitory control reduces mechanical allodynia in preclinical models	Provides a strong mechanistic basis, but clinical translation remains early [27]
Synaptic vesicle release	Spinal cord injury‐related neuropathic pain models	SV2A modulation, including brivaracetam	May reduce abnormal synaptic vesicle release in injured spinal circuits	SV2A modulation reduces neuropathic pain‐like behavior in preclinical models	Supports clinical testing in spinal cord injury‐related neuropathic pain [147
AAK1 pathway	Diabetic and peripheral neuropathic pain models	AAK1 inhibition, including LX9211	Modulates endocytosis‐related neuronal signaling and pain transmission	AAK1 inhibition reduces neuropathic pain‐like behavior in animal models	Supports further clinical trials in painful diabetic neuropathy [150]
SSTR4 pathway	Inflammatory and neuropathic pain models	SSTR4 agonists	Activates somatostatin receptor 4 signaling and reduces pain transmission	SSTR4 agonists show analgesic effects in preclinical pain models	Supports early clinical testing, but human evidence is still limited [161]
P2X3 pathway	Peripheral nerve injury and diabetic neuropathy‐related models	P2X3 antagonists	Reduces purinergic activation of peripheral sensory neurons	P2X3 antagonism reduces sensory neuron activation in preclinical pain models	Clinical results in diabetic neuropathic pain have been mixed [162]
Glia‐neuron neuroimmune signaling	Peripheral nerve injury and neuroinflammatory pain models	Locus coeruleus–spinal cord noradrenergic signaling, astrocytic mGluR5, and cytokine/chemokine pathways	Limits microglial and astrocytic activation, modulates inflammatory mediator release, and normalizes glia‐associated glutamatergic signaling	Pathway‐ or cell‐selective modulation reduced neuronal hyperexcitability and pain‐like behavior in nerve‐injury models	Identifies glia–neuron signaling as a potential mechanism‐informed target, although cell‐state‐specific biomarkers and clinical validation remain necessary [10, 151]
Circuit‐level modulation	Rodent pain models and translational stimulation models	SCS, rTMS, tDCS, DBS, temporal interference stimulation, and focused ultrasound	Regulates spinal gating, thalamo‐cortical processing, affective pain circuits, or descending control	Neuromodulation can reduce pain‐like behavior and alter pain‐related network activity	Supports imaging‐guided target selection and response monitoring [148, 149, 164, 167]

Abbreviations: AAK1, adaptor‐associated kinase 1; DBS, deep brain stimulation; DRG, dorsal root ganglion; KCC2, potassium‐chloride cotransporter 2; LX9211, pilavapadin; NaV1.7, NaV1.8, and NaV1.9, voltage‐gated sodium channel subtypes 1.7, 1.8, and 1.9; NMDA, N‐methyl‐D‐aspartate; P2X3, purinergic P2X receptor 3; rTMS, repetitive transcranial magnetic stimulation; SCS, spinal cord stimulation; SSTR4, somatostatin receptor 4; SV2A, synaptic vesicle glycoprotein 2A; tDCS, transcranial direct current stimulation; α2δ‐1, alpha‐2‐delta‐1 auxiliary subunit of voltage‐gated calcium channels.

**TABLE 4:** Recent and ongoing clinical trials by pathway and mechanism of action.

Pathway	Agent or device	NCT No.	Status and phase	Objective	Preliminary findings or current interpretation
Nav1.8 inhibition	Suzetrigine/VX‐548	NCT05660538	Completed, Phase 2	To evaluate the efficacy and safety of VX‐548 in painful diabetic peripheral neuropathy	Phase 2 data support further development in painful diabetic neuropathy. This pathway is clinically important because it targets peripheral nociceptor firing without opioid activity [155, 156]
Nav1.8 inhibition	Suzetrigine	NCT06628908	Recruiting, Phase 3	To evaluate the efficacy, safety, and tolerability of suzetrigine in painful diabetic peripheral neuropathy	No final published result is available yet. This trial is important because it tests Nav1.8 inhibition in a large DPN population [156]
Nav1.8 inhibition	Suzetrigine	NCT07231419	Recruiting, Phase 3	To evaluate the efficacy, safety, and tolerability of suzetrigine in painful diabetic peripheral neuropathy	No final published result is available yet. This trial provides confirmatory evidence for the same pathway [156]
Nav1.8 inhibition	Suzetrigine	NCT06696443	Phase 3, open‐label long‐term study	To evaluate long‐term safety, tolerability, and effectiveness in painful diabetic peripheral neuropathy	No final published result is available yet. This study focuses on long‐term use [156]
AAK1 inhibition	LX9211/Pilavapadin	NCT04455633	Completed, Phase 2	To evaluate efficacy, safety, and pharmacokinetics in diabetic peripheral neuropathic pain	Published Phase 2 data showed pain reduction with LX9211, supporting AAK1 inhibition as a promising pathway [150]
AAK1 inhibition	LX9211/Pilavapadin	NCT04662281	Completed, Phase 2	To evaluate efficacy in postherpetic neuralgia	Available reports suggest that results differ by indication. This pathway should be interpreted as promising but not yet established [150]
SSTR4 activation	LY3356050	NCT06074562	Completed, Phase 2	To determine safety and efficacy versus placebo in diabetic peripheral neuropathic pain	No peer‐reviewed final result was found. This pathway remains at an early clinical stage [161]
Chemokine signaling	GSK3858279	NCT05838755	Phase 2	To evaluate efficacy, safety, tolerability, pharmacokinetics, and target engagement in chronic diabetic peripheral neuropathic pain	No final published result was found. The trial tests CCL17 blockade as a neuroimmune treatment approach [163]
P2X3 antagonism	Eliapixant/BAY1817080	NCT04641273	Completed, Phase 2a	To evaluate efficacy and safety in diabetic neuropathic pain	The PUCCINI study did not meet its primary endpoint and did not proceed to Phase 2b [162]
SV2A modulation	Brivaracetam	NCT04379011	Completed, Phase 1/2 pilot	To assess feasibility, safety, and tolerability in spinal cord injury‐related neuropathic pain	Pilot data support feasibility and tolerability, but the sample size was small [147]
SV2A modulation	Brivaracetam	NCT05639946	Completed, Phase 3 in registry summaries	To evaluate efficacy in spinal cord injury‐related neuropathic pain	Full peer‐reviewed efficacy data should be checked before final submission [147]
NMDA/AMPA‐related plasticity	(2R,6R)‐hydroxynorketamine	NCT05864053	Phase 1/2	To compare hydroxynorketamine, ketamine, and saline in neuropathic pain of the extremities	No final published clinical result was found. Preclinical evidence supports further testing [160]
Cortical modulation	rTMS	NCT04833660	Clinical trial in painful diabetic neuropathy	To test high‐frequency rTMS over M1 for lower‐extremity diabetic neuropathic pain	Published clinical work suggests short‐term benefit of M1 rTMS in painful diabetic neuropathy [149]
Focused ultrasound neuromodulation	FUS/LIFU/MRgFUS	NCT04485208, NCT05122403, NCT06906211, NCT07226648	Active or recent early‐stage/device studies	To test ultrasound‐based modulation of pain‐related brain regions	Evidence remains early. These studies fit the imaging‐guided treatment framework because ultrasound targeting often depends on MRI or functional imaging [148, 167]

Abbreviations: AAK1, adaptor‐associated kinase 1; AMPA, α‐amino‐3‐hydroxy‐5‐methyl‐4‐isoxazolepropionic acid; CCL17, C–C motif chemokine ligand 17; DPN, diabetic peripheral neuropathy; FUS, focused ultrasound; LIFU, low‐intensity focused ultrasound; M1, primary motor cortex; MRI, magnetic resonance imaging; MRgFUS, magnetic resonance‐guided focused ultrasound; NCT, ClinicalTrials.gov identifier; NMDA, N‐methyl‐D‐aspartate; NaV1.8, voltage‐gated sodium channel subtype 1.8; P2X3, purinergic P2X receptor 3; rTMS, repetitive transcranial magnetic stimulation; SSTR4, somatostatin receptor 4; SV2A, synaptic vesicle glycoprotein 2A; VX‐548, suzetrigine.

### 6.3 Imaging‐Guided Neuromodulation

The pathophysiological mechanisms behind neuropathic pain involve all types of abnormalities in the central and peripheral nervous systems, such as ion channel problems, neurotransmitter imbalances, neuroinflammation, and changes in neural circuit plasticity. Different neuromodulation techniques work by targeting these pathways to relieve pain, while imaging tools play a key role in precise targeting, real‐time monitoring, and evaluating how well the treatment works.

Spinal cord stimulation (SCS) is one of the most commonly used neuromodulation techniques for treating intractable neuropathic pain. It delivers high‐frequency stimulation to the dorsal column via implanted electrodes, which activates Aβ fibers and suppresses nociceptive signal transmission to the thalamus through the gate control mechanism [169]. Repetitive transcranial magnetic stimulation (rTMS) applies a time‑varying magnetic field to specific brain regions, modulating cortical excitability and neural plasticity. Its analgesic effect may arise from M1 regulating the descending inhibitory system via the cortex–thalamus–spinal cord pathway [170]. The combination of TMS with EEG can assess cortical responses to stimulation. A study of 21 patients with neuropathic pain and 21 healthy controls found differences in responses within sensory, emotional, and attention‐related cortical regions. Some measures were associated with pain‐related negative emotions [171].

The ventral posterolateral nucleus (VPL) is a key relay nucleus in the thalamus that transmits somatosensory signals. It projects nociceptive information from the spinothalamic tract to the primary somatosensory cortex. Transcranial temporal interference stimulation (tTIS) targets the VPL noninvasively, generating interference electric fields that activate deep neurons and mimic the analgesic effects of deep brain stimulation (DBS). One study compared VPL‐targeted temporal interference stimulation with high‐definition alternating current stimulation over the sensorimotor cortex during sustained experimental pain [172]. A retrospective multicenter study examined DBS for central poststroke pain. The final analysis included 39 patients with available imaging data. Fourteen patients reported at least 50% pain relief at 12 months. Stimulation sites in responders had lower structural connectivity to sensory cortical areas than those in nonresponders [173]. In terms of imaging‑based prediction, machine learning models using electroencephalography (EEG) can forecast the response to transcranial direct current stimulation (tDCS). One observational study in patients with neuropathic pain after SCI aimed to develop an artificial intelligence model based on baseline EEG signals and clinical assessment data to predict the success rate of tDCS treatment. This work provides a new technical route for individualized tDCS interventions [174].

### 6.4 Imaging‐Guided Monitoring and Ultrasound‐Guided Therapy

Noninvasive focused ultrasound (FUS) technology has opened a completely new direction for the neuromodulation of neuropathic pain. Among the various approaches, low‐intensity transcranial focused ultrasound (LIFU) stands out as a highly promising analgesic modality due to its unique advantages of noninvasiveness, high spatial resolution, and the ability to penetrate the skull [175]. Human studies have reported both increased and decreased neural responses after low‐intensity transcranial focused ultrasound. These effects vary with the stimulation parameters and target region [176, 177]. In mice, ultrasound stimulation of the primary somatosensory cortex or insula changed pain‐related behavior [178].

Ultrasound‐guided dry needling of trigger points may help relieve pain in patients with postherpetic neuralgia and coexisting myofascial pain syndrome when combined with other needling techniques [179]. Bubnov reported the use of ultrasound‐guided dry needling for myofascial pain in 2010. Real‐time ultrasound helps clinicians visualize the target tissue and guide needle placement [180]. A review also proposed combining ultrasound, MRI, PET, and SPECT with electromyography to support personalized pain assessment and management.

In terms of peripheral neuroimaging monitoring, FUS may influence multiple mechanisms relevant to neuropathic pain management, such as ion channel regulation, glutamatergic neurotransmission, cerebral blood flow, inflammation and neurotoxicity, neuronal morphology and survival, nerve regeneration, and myelination [148]. The use of elastography‐based virtual tissue imaging quantification (VTIQ) has enabled noninvasive, quantitative assessment of sciatic neuropathy in patients with type 2 diabetes mellitus, representing an important contribution to peripheral neuroimaging monitoring. For the first time, the combined application of nerve elastic modulus, cross‑sectional area, and nerve conduction velocity significantly improved the diagnostic efficacy for diabetic polyneuropathy, providing a clinical imaging biomarker that simultaneously reflects nerve structure, stiffness, and functional status, thus overcoming the limitations of conventional ultrasound (which only visualizes morphology) and electrophysiology (which only assesses function) [129].

Researchers used functional ultrasound to measure brain hemodynamic responses to peripheral focused ultrasound stimulation in mice. The responses varied with stimulation intensity, and functional connectivity differed between healthy mice and mice with neuropathic pain. These findings support further study of ultrasound imaging for monitoring experimental neuromodulation [181]. The mechanism of DN‑US in treating mixed myofascial and neuropathic pain is not simply local muscle relaxation but rather a systemic process involving peripheral nerve morphological remodeling, improvement of local microcirculation, spinal‑level reflex modulation, and changes in whole–brain functional network connectivity [182]. Through dynamic monitoring of peripheral nerve ultrasound parameters and central imaging biomarkers before and after treatment, the effects of peripheral local interventions can be linked to plastic changes at the central nervous system level, thereby establishing a causal relationship from peripheral pain generators and central sensitization to clinical outcomes [183].

## 7 Conclusion and Prospects

Neuropathic pain is not only a symptom caused by nerve injury. It is a network disorder supported by molecular imbalance and abnormal circuit activity. Excess glutamate, weakened GABAergic and glycinergic inhibition, altered monoaminergic control, and persistent glial activation can act together to maintain pain. These changes increase excitation, weaken descending inhibition, and promote neuroinflammation. They also help explain why pain can persist even after the original nerve injury becomes less active.

These molecular changes affect several pain‐related circuits. The ACC–VTA circuit links pain with motivation and reward. Thalamocortical relays support sensory amplification. The parabrachial–amygdala pathway links nociceptive input with negative emotion. The amygdala–PFC–PAG pathway affects descending pain control. The hippocampus–habenula–VTA axis and cerebello–thalamo–cortical loops may further shape pain memory, prediction, and behavioral responses. These circuits do not work alone. Instead, they form a broad pain network that connects sensory, emotional, cognitive, and autonomic processes.

Multimodal imaging provides a practical way to study these mechanisms in patients. MRI and DTI can assess structural integrity and white matter changes. fMRI and fNIRS can measure functional connectivity. MRS can assess glutamate and GABA levels and may reflect the excitation–inhibition balance. PET can measure receptors, transporters, glial activation, and other molecular targets. These tools can help match patients with mechanism‐based treatments. They can also help monitor whether a treatment has reached its biological target, such as reduced glial activation on TSPO‐PET or improved excitation–inhibition balance on MRS.

Several challenges still limit clinical translation. Patients with neuropathic pain differ in disease cause, pain duration, sensory phenotype, mood, sleep, and previous treatment exposure. These differences reduce the value of broad group‐level findings. Animal models also cannot fully reproduce the complexity of human neuropathic pain. Future studies should therefore enroll patients according to dominant mechanisms, such as peripheral ion‐channel hyperexcitability, spinal disinhibition, neuroimmune activation, or impaired descending control. Multicenter studies should standardize imaging protocols, clinical outcomes, and data analysis. Longitudinal registries should combine imaging, sensory testing, electrophysiology, clinical scales, and molecular data. Machine learning may help predict treatment response, but these models need external validation. Closed‐loop neuromodulation guided by real‐time imaging or electrophysiological signals may become an important future direction. The main goal is to move neuropathic pain care from broad treatment selection toward mechanism‐based precision medicine.

## Author Contributions

Yaling Dai, Mengquan Tan, and Man Yuan contributed equally to the conception and design of the review; performed the literature search; screened the articles; extracted and synthesized the data; and drafted the initial manuscript. Huixian Zhou participated in the literature search and data extraction, analyzed and interpreted the data, organized the findings, and assisted with figure and table preparation. Qin Xie and Xinran He contributed to the organization and interpretation of the extracted data and critically revised the manuscript for important intellectual content. Jiling Zeng, Yixin Yang, and Siyuan Song conceived and supervised the study, resolved discrepancies in study selection and data interpretation, and critically revised the manuscript. All the authors have read and approved the final manuscript.

## Funding

This work was supported by the National Natural Science Foundation of China (grant no. 82505722), the Fujian Provincial Natural Science Foundation (grant no. 2025J08106) and the National Natural Science Foundation of China (grant no. 82474616).

## Ethics Statement

The authors have nothing to report.

## Conflicts of Interest

The authors declare no conflicts of interest.

## Data Availability

Data and materials are available from the corresponding authors upon reasonable request.
